# Dynamic Variability of Sodium and Potassium Predicts Mortality in Critically Ill Patients With Ischemic Stroke: Evidence From Two Critical Care Cohorts

**DOI:** 10.1002/brb3.71626

**Published:** 2026-09-04

**Authors:** Jian Zhang, Wei Zhang, Bin Zeng, Linfeng Xie, Jianjun Zhu

**Affiliations:** ^1^ Department of Emergency and Critical Care Medicine The Second Affiliated Hospital of Soochow University Suzhou People's Republic of China; ^2^ Department of Cardiology The First Affiliated Hospital of Chongqing Medical University Chongqing People's Republic of China

**Keywords:** ICU mortality, ischemic stroke, MIMIC‐IV, NWICU, potassium variability, sodium variability

## Abstract

**Background:**

Ischemic stroke (IS) is one of the leading causes of death and long‐term disability worldwide. Electrolyte fluctuations, particularly in sodium and potassium, may play a critical role in the mortality of IS.

**Methods:**

This retrospective cohort study utilized the MIMIC‐IV database and NWICU to examine the association between sodium and potassium variability (coefficient of variation [CV]) and mortality in IS patients. A total of 3266 IS patients were included. The study focused on 30‐, 90‐, and 360‐day all‐cause mortality. Patients were stratified into quartiles based on CV values for sodium and potassium variability. Multivariable Cox proportional‐hazards models were used to assess associations. Cumulative incidence was estimated using Kaplan–Meier methods. Restricted cubic spline (RCS) analyses were conducted to examine the associations of sodium and potassium CVs with the primary outcomes. Sensitivity analyses further examined the impact of electrolyte levels and variability on mortality.

**Results:**

In this study, 2593 patients from MIMIC‐IV and 673 patients from NWICU were analyzed by sodium and potassium fluctuation quartiles. Sodium and potassium variability were significantly associated with mortality at 30‐day, 90‐day, and 360‐day intervals, with continuous sodium variability showing HRs of 1.22 (30‐day), 1.18 (90‐day), and 1.17 (360‐day), and Q4 significantly increasing risk (HR = 1.78, 1.51, 1.49, respectively). In NWICU, Q4 sodium variability was an independent risk factor for 30‐day mortality (HR = 4.04, *p* < 0.001). Sensitivity analyses confirmed that both high and low sodium and potassium levels were associated with increased mortality risks.

**Conclusions:**

Fluctuations in sodium and potassium levels were strongly linked to increased short‐term and long‐term mortality risk in intensive care unit (ICU) patients. Patients with high variability in both electrolytes had the worst prognosis.

## Graphical abstract

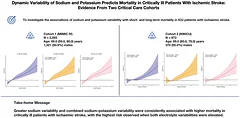

## 1 Introduction

Ischemic stroke (IS) is one of the leading causes of death and long‐term disability worldwide (Feske 2021). When an ischemic stroke occurs, brain tissue becomes deprived of oxygen due to a disruption in blood supply, leading to damage or death of brain cells (Ho and Powers 2025). For patients with ischemic stroke, early intervention and active treatment can significantly improve prognosis, and management in the intensive care unit (ICU) plays a crucial role in this process (Salih et al. 2022).

Sodium–potassium imbalance is commonly observed in patients with IS, and its dynamic fluctuation (i.e., variability in sodium and potassium levels) is believed to be closely related to the pathophysiological mechanisms of the stroke (Shehjar et al. 2023). Sodium and potassium are essential electrolytes in the body, playing a crucial role in maintaining normal cellular function, regulating fluid balance, and modulating neural electrical activity. Ischemic stroke can lead to damage of the blood–brain barrier (Jiang et al. 2018), allowing inflammatory mediators (Zhu et al. 2022), plasma proteins, and other harmful substances to enter brain tissue (Jia et al. 2025), further exacerbating brain edema and injury. Research has shown that disruption of the blood–brain barrier may lead to an imbalance in sodium and potassium levels, intensifying electrolyte fluctuations (Kadry et al. 2020). As these changes occur, stroke patients may face more complex electrolyte management issues, particularly in ICU settings (Martha et al. 2020). Monitoring and appropriately managing sodium–potassium variability can not only help assess the condition of ischemic stroke patients but also provide important guidance for early intervention and individualized treatment (Herpich and Rincon 2020).

Previous studies have been limited to the impact of individual electrolyte markers on stroke, such as the association between hyponatremia and hypernatremia with higher mortality in stroke patients (Yuen et al. 2022). Furthermore, existing research mainly relies on single‐point electrolyte concentration measurements. One study indicated that lower serum potassium levels were independently associated with an increased risk of stroke recurrence in patients with AIS or TIA (Wang et al. 2022). However, to date, no study has systematically evaluated the impact of the temporal dynamics of sodium and potassium homeostasis on mortality in IS patients. These dynamic changes may better reflect the severity of the disease and prognosis.

Therefore, we used the MIMIC‐IV database to quantify the association between sodium and potassium concentration fluctuations, measured by the coefficient of variation (CV) threshold, and the short‐term (30‐day) and long‐term (90‐day and 360‐day) all‐cause mortality in IS ICU patients. By further exploring the prognosis based on the cutoff values of sodium and potassium variability, we aim to provide actionable insights for electrolyte management in neurocritical care.

## 2 Methods

### 2.1 Data Source

The databases used in this study were obtained from the MIMIC‐IV (v3.1) and NWICU (v0.1.0). MIMIC‐IV database, released on December 19, 2024 (https://mimic‐iv.mit.edu/) (Johnson et al. 2023), was developed by MIT and Beth Israel Deaconess Medical Center, and includes de‐identified ICU patient data from admission to discharge. NWICU is a critical care unit (ICU) research database jointly compiled by Northwestern University (NU) and Northwestern Memorial Health Care (NMHC) based on authentic clinical data from intensive care units during 2020–2022, and publicly available on the MIT/PhysioNet platform. The study was approved by the Institutional Review Boards of both institutions (BIDMC: 2001‐P‐001699/14; MIT: 0403000206). As the data are de‐identified and publicly accessible, no additional ethical approval or informed consent was required. One of the authors (Linfeng Xie) holds a Human Subjects Research Training Certificate (no. 57983166).

### 2.2 Study Subjects

This retrospective cohort study used two large critical care databases (MIMIC‐IV v3.1 and NWICU). In MIMIC‐IV v3.1, 94,458 ICU admissions were screened, and 10,488 patients with cerebral infarction were identified. After excluding repeated admissions (*n* = 1244), patients not actually admitted to the ICU (*n* = 6260), those with fewer than three sodium/potassium measurements (*n* = 157), age < 18 years (*n* = 0), ICU length of stay < 1 day (*n* = 190), and patients with cardiovascular abnormalities in sodium and potassium levels (*n* = 44), 2593 patients remained for final analysis. In the NWICU database, 28,612 ICU admissions were screened and 2673 patients with cerebral infarction admitted to the ICU were identified. After excluding repeated admissions (*n* = 1267), cases with fewer than three sodium/potassium measurements (*n* = 703), patients < 18 years (*n* = 0), ICU stay < 1 day (*n* = 6), and patients with cardiovascular abnormalities in sodium and potassium levels (*n* = 24), 673 patients were retained. Overall, 3266 patients from both databases were included in the final cohort (Figure S1).

### 2.3 Data Extraction

Data extraction was performed using Navicat Premium version 16.3.2 to query the MIMIC‐IV and NWICU database. Comprehensive clinical data for all eligible IS patients were systematically retrieved. The clinical variables included demographic information (age, sex, and race), vital signs (heart rate, systolic blood pressure [SBP], diastolic blood pressure [DBP], and respiratory rate), and clinical comorbidities categorized according to ICD codes (hypertension, diabetes mellitus, chronic obstructive pulmonary disease, congestive heart failure, malignant tumors [in MIMIC‐IV], chronic kidney disease, and liver disease). Additionally, laboratory parameters recorded within the first 24 h of ICU admission were collected, including platelets, hemoglobin, white blood cells, chloride, calcium, initial sodium, initial potassium, creatinine, and glucose. Clinical scoring systems, such as the Acute Physiology Score III (APS‐III) and Glasgow Coma Scale (GCS) score in MIMIC‐IV, were also included. Interventions within the first 24 h following ICU admission, such as renal replacement therapy (hemodialysis in NWICU), invasive mechanical ventilation, potassium supplementation, sodium supplementation, thrombolysis (in MIMIC‐IV), antiplatelet drugs, warfarin, and furosemide, were documented. For patients with multiple measurements, the first recorded value within the initial 24 h of ICU admission was extracted. Special attention was given to data points where serum sodium and potassium levels were measured more than three times throughout the entire ICU admission period.

### 2.4 Exposure and Patient Definition

The mean serum sodium and potassium levels were calculated by averaging all serum sodium and potassium measurements taken throughout the patient's ICU stay. To assess the variability of sodium and potassium levels, the CV was computed for each patient based on all sodium and potassium measurements recorded after ICU admission. The CV was calculated as (standard deviation / mean) × 100%. Patients with ischemic stroke encompass two disease types: cerebral infarction and transient ischemic attack (Hilkens et al. 2024).

### 2.5 Outcomes

The primary outcomes of the study were all‐cause mortality at 30, 90, and 360 days following ICU admission. These endpoints, hereafter referred to as “30‐day, 90‐day, and 360‐day mortality,” represent any death occurring within the specified timeframes starting from the date of the initial ICU admission, regardless of whether the patient remained in the ICU or had been discharged from the hospital. These timeframes were selected to provide comprehensive insights into both the short‐term and long‐term survival outcomes of ischemic stroke patients managed in the intensive care setting.

### 2.6 Statistical Analysis

The missing data rates for each variable are presented in Table S1. For variables with missingness < 20%, multiple imputation was performed using the “mice” package in R. This method generated several datasets, imputed missing values multiple times, and combined the results to maintain data integrity. Normality tests indicated that all continuous variables were non‐normally distributed, so they are reported as medians with interquartile ranges (IQR). Group differences were assessed using Kruskal–Wallis tests. Categorical variables are expressed as counts and percentages, with comparisons made using Fisher's exact test or the Chi‐squared test.

Participants were stratified into four groups (Q1–Q4) according to the quartiles of sodium and potassium variability (CV). In MIMIC‐IV, sodium variability quartiles were defined as follows: Q1 < 0.94; 0.94 ≤ Q2 < 1.43; 1.43 ≤ Q3 < 2.04; Q4 ≥ 2.04. Potassium variability quartiles were defined as follows: Q1 < 5.25; 5.25 ≤ Q2 < 7.90; 7.90 ≤ Q3 < 11.62; Q4 ≥ 11.62. In NWICU, sodium variability quartiles were defined as follows: Q1 < 0.95; 0.95 ≤ Q2 < 1.50; 1.50 ≤ Q3 < 2.36; Q4 ≥ 2.36. Potassium variability quartiles were defined as follows: Q1 < 5.18; 5.18 ≤ Q2 < 7.90; 7.90 ≤ Q3 < 10.88; Q4 ≥ 10.88. Before fitting the multivariable Cox proportional hazards models, we assessed multicollinearity among covariates using variance inflation factors (VIFs). A VIF < 5 was considered to indicate no severe multicollinearity (Table S2). Two multivariable Cox proportional‐hazards models with incremental adjustment were fitted to evaluate associations between quartiles of sodium and potassium CVs and ICU all‐cause mortality at 30, 90, and 360 days in patients with ischemic stroke, using Q1 as the reference. Hazard ratios (HRs) and 95% confidence intervals (CIs) were reported for Q2–Q4. Model I: unadjusted. Model II: adjusted for age, sex, race, heart rate, systolic and diastolic blood pressure, respiratory rate, hypertension, diabetes mellitus, chronic pulmonary disease, acute myocardial infarction, congestive heart failure, malignancy, chronic kidney disease, liver disease, platelet count, hemoglobin, white blood cell count, chloride, calcium, creatinine, glucose, clinical severity scores (APS III and GCS in MIMIC‐IV), renal replacement therapy, mechanical ventilation, potassium supplementation, sodium supplementation, thrombolysis, antiplatelet therapy, warfarin, and furosemide. Maximally selected rank statistics were used to determine the optimal cut‐off values of sodium and potassium CVs for 30‐, 90‐, and 360‐day mortality; these thresholds were then applied Cox models to evaluate the joint effects of sodium and potassium on the three mortality outcomes. We defined four joint‐exposure groups: low sodium variability and low potassium variability; low sodium variability and high potassium variability; high sodium variability and low potassium variability; and high sodium variability and high potassium variability.

In addition, cumulative incidence of ICU mortality at 30, 90, and 360 days was estimated using the Kaplan–Meier method, and between‐group differences were assessed with the log‐rank test. Within the Cox proportional‐hazards framework, restricted cubic spline (RCS) analyses were conducted to examine the associations of sodium and potassium CVs with the primary outcomes. Nonlinear relationships and overall significance were summarized by *p* values.

We performed several sensitivity analyses: (i) to further characterize the impact of electrolyte fluctuations, mean serum sodium and potassium levels were calculated based on multiple measurements taken after ICU admission, patients were classified by mean sodium level into normonatremia (135–145 mmol/L), hyponatremia (< 135 mmol/L), and hypernatremia (> 145 mmol/L), and by mean potassium level into normokalemia (3.5–5.5 mmol/L), hypokalemia (< 3.5 mmol/L), and hyperkalemia (> 5.5 mmol/L); using the normal‐level groups as the reference, we examined associations with 30‐, 90‐, and 360‐day ICU mortality. (ii) To further delineate the effects of sodium and potassium variability on mortality, we evaluated quartiles of their CVs in relation to ICU mortality and in‐hospital mortality. (iii) To address potential confounding by malignancy, we excluded patients with malignant tumor and repeated analyses of quartiles of sodium and potassium variability and of mean sodium and potassium levels for 30‐, 90‐, and 360‐day ICU mortality. As there were no patients with malignant tumor comorbidities among IS patients in the NWICU, we conducted this analysis solely within the MIMIC‐IV cohort. (iv) To rigorously address potential residual confounding from complex therapeutic interventions and severe metabolic derangements, we additionally adjusted for variables extracted within the first 24 h of ICU admission, including fluid management (24‐h net fluid balance), calculated serum osmolarity, glycemic control (glucose CV and insulin), glucose‐corrected sodium levels, sepsis, vasopressor, pH, and lactate.

All statistical analyses were performed using SPSS Statistics (v27), STATA (v18), and R software (v4.4.3). A two‐tailed *p* value < 0.05 was considered statistically significant.

## 3 Results

### 3.1 Baseline Characteristics

Baseline characteristics of the overall study populations from MIMIC‐IV and NWICU are summarized in Table 1. In the MIMIC‐IV cohort, baseline characteristics stratified by quartiles of sodium variability and potassium variability are presented in Tables S3 and S4, respectively. In the NWICU cohort, corresponding baseline characteristics stratified by quartiles of sodium variability and potassium variability are shown in Tables S5 and S6.

**TABLE 1:** Baseline characteristics of patients in the MIMIC‐IV and NWICU.

Characteristics	MIMIC‐IV (*n* = 2593)	NWICU (*n* = 673)
Age, years	69.0 (59.0, 80.0)	69.0 (60.0, 78.0)
Male, *n* (%)	1321 (50.9)	370 (55.0)
Race, *n* (%)		
White	1497 (57.7)	433 (64.3)
Black	225 (8.7)	104 (15.5)
Hispanic	76 (2.9)	—
Asian	78 (3.0)	2 (0.3)
Others	717 (27.7)	134 (19.9)
Vital signs		
Heart rate, bpm	80.4 (70.6, 91.6)	84.0 (73.0, 98.0)
Systolic blood pressure, mmHg	128.5 (115.9, 142.6)	138.0 (119.0, 160.0)
Diastolic blood pressure, mmHg	67.5 (59.7, 77.2)	74.0 (63.0, 87.0)
Respiratory rate, bpm	18.8 (16.9, 21.4)	20.0 (16.0, 24.0)
Comorbidities, *n* (%)		
Hypertensive	1331 (51.3)	145 (21.5)
Diabetes mellitus	563 (21.7)	89 (13.2)
Chronic pulmonary disease	157 (6.1)	18 (2.7)
Acute myocardial infarction	254 (9.8)	28 (4.2)
Congestive heart failure	628 (24.2)	40 (5.9)
Malignant tumor	247 (9.5)	0 (0.0)
Chronic kidney disease	432 (16.7)	40 (5.9)
Liver disease	105 (4.0)	17 (2.5)
Laboratory tests		
Platelets, K/uL	207.0 (157.0, 270.0)	209.0 (161.0, 268.8)
Hemoglobin, g/dL	11.6 (9.8, 13.3)	12.1 (10.0, 13.6)
White blood cells, K/uL	10.6 (8.0, 14.2)	10.2 (7.8, 13.9)
Chloride, mmol/L	104.0 (101.0, 107.0)	106.0 (102.0, 109.0)
Calcium, mg/dL	8.6 (8.1, 9.0)	8.6 (8.2, 9.0)
Initial sodium, mEq/L	139.0 (136.0, 142.0)	139.0 (136.0, 141.0)
Initial potassium, mEq/L	4.1 (3.7, 4.5)	3.9 (3.6, 4.2)
Mean sodium, mEq/L	139.8 (137.3, 142.3)	139.8 (137.4, 142.3)
Mean potassium, mEq/L	4.0 (3.8, 4.3)	3.9 (3.7, 4.1)
Creatinine, mg/dL	0.9 (0.7, 1.3)	106.0 (102.0, 109.0)
Glucose, mg/dL	128.0 (104.0, 165.0)	134.0 (110.0, 174.5)
Clinical scoring		
Acute Physiology Score III	39.0 (29.0, 53.0)	—
Glasgow Coma Scale score	15.0 (14.0, 15.0)	—
Intervention, *n* (%)		
Renal replacement therapy/hemodialysis	43 (1.7)	7 (1.0)
Invasive mechanical ventilation	1160 (44.7)	150 (22.3)
Potassium supplementation	610 (23.5)	140 (20.8)
Sodium supplementation	1769 (68.2)	294 (43.7)
Thrombolysis	53 (2.0)	—
Antiplatelet drugs	925 (35.7)	227 (33.7)
Warfarin	153 (5.9)	6 (0.9)
Furosemide	166 (6.4)	48 (7.1)
Clinical outcomes		
30‐day mortality, *n* (%)	638 (24.6)	109 (16.2)
90‐day mortality, *n* (%)	812 (31.3)	128 (19.0)
360‐day mortality, *n* (%)	976 (37.6)	151 (22.4)
ICU length of stay, day	4.2 (2.2, 8.0)	4.0 (2.8, 6.8)
Length of hospital stay, day	9.9 (5.8, 17.8)	8.8 (5.2, 15.9)

Overall, in MIMIC‐IV, patients in Q4 exhibited significantly higher mean age, heart rate, systolic blood pressure, diastolic blood pressure, and respiratory rate compared to other quartiles (*p* < 0.001). Prevalence of diabetes mellitus, hypertension, and chronic kidney disease was also higher in Q4 (*p* < 0.05). The Q4 group exhibited the highest APS‐III scores (*p* < 0.001) and significantly higher rates of mechanical ventilation and sodium supplementation compared to other groups (*p* < 0.001). Regarding clinical outcomes, the Q4 group exhibited the highest 30‐day mortality rate (26.7% vs. 34.1%, *p* < 0.001), along with the longest ICU and total hospitalization durations (*p* < 0.001). In NWICU database, for sodium variability, there were no significant differences in age, sex, race, or most comorbidities between quartiles (*p* > 0.05). For potassium variability, age and race were similar across quartiles, but the proportion of male patients decreased from Q1 to Q4 (*p* < 0.001).

### 3.2 The Association Between Sodium and Potassium Variability and 30/90/360‐Day Mortality in MIMIC‐IV

**FIGURE 1:**
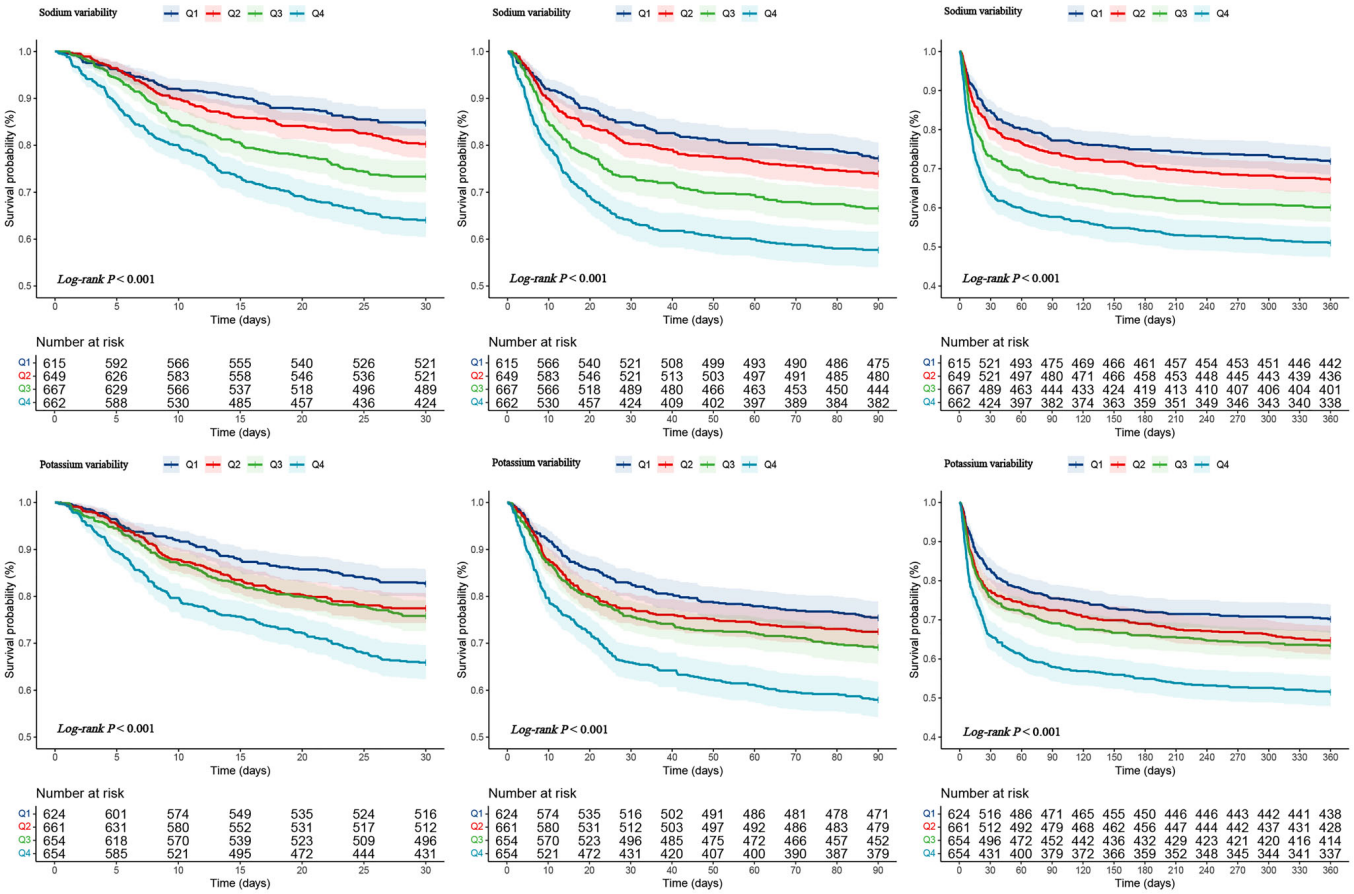
Kaplan–Meier survival curves between sodium and potassium variability and 30‐day, 90‐day, 360‐day mortality in the MIMIC‐IV.

**FIGURE 2:**
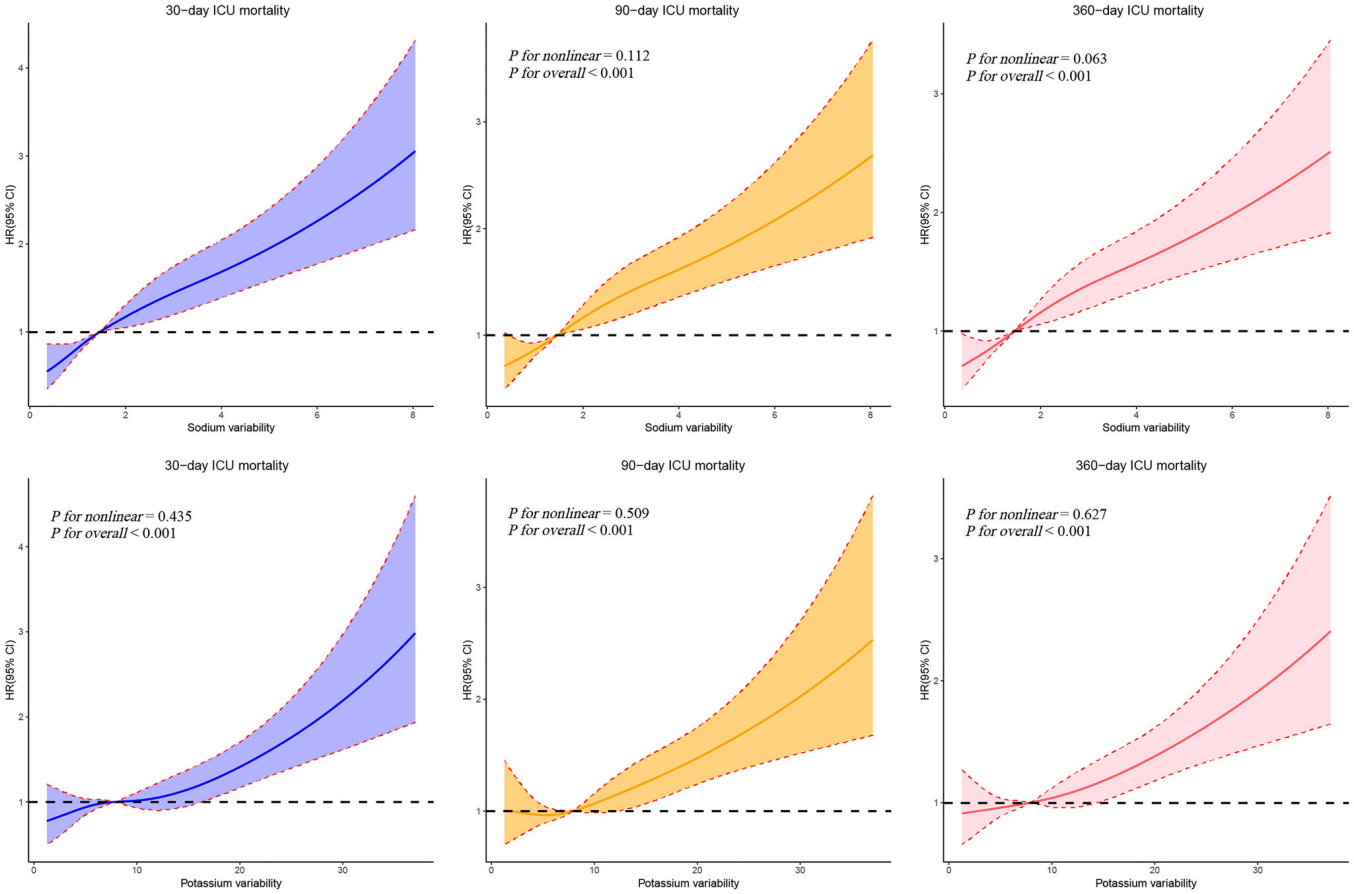
The restricted cubic spline for the relationships between sodium and potassium variability and 30‐day, 90‐day, and 360‐day mortality in the MIMIC‐IV.

Table 2 presents the association between sodium and potassium variability and 30‐day, 90‐day, and 360‐day mortality. Collinearity diagnostics confirmed that the VIFs for all included covariates were below 5.0. In Model II, for sodium variability, continuous sodium variability showed a significant association with 30‐day (HR = 1.22, 95% CI: 1.16–1.28, *p* < 0.001), 90‐day (HR = 1.18, 95% CI: 1.13–1.24, *p* < 0.001), and 360‐day (HR = 1.17, 95% CI: 1.12–1.22, *p* < 0.001) mortality. For the quartiles of sodium variability, compared to Q1, Q4 was an independent risk factor for 30‐day (HR = 1.78, 95% CI: 1.39–2.28, *p* < 0.001), 90‐day (HR = 1.51, 95% CI: 1.22–1.87, *p* < 0.001), and 360‐day (HR = 1.49, 95% CI: 1.23–1.81, *p* < 0.001) mortality. Kaplan–Meier analyses revealed significant 30‐day, 90‐day, and 360‐day mortality differences across sodium variability, all log‐rank *p* < 0.001 (Figure 1). Patients in the Q4 exhibited significantly worse outcomes compared to Q1. RCS analyses (Figure 2) demonstrated a linear association between sodium variability and 30‐day (*p* for nonlinear = 0.112, *p* for overall < 0.001), 90‐day (*p* for nonlinear = 0.112, *p* for overall < 0.001), and 360‐day mortality (*p* for nonlinear = 0.063, *p* for overall < 0.001).

**TABLE 2:** The association between sodium and potassium variability and mortality in the MIMIC‐IV.

	Sodium variability			Potassium variability				
	Model I		Model II		Model I		Model II	
	HR (95% CI)	*p*	HR (95% CI)	*p*	HR (95% CI)	*p*	HR (95% CI)	*p*
**30‐day mortality**								
Continuous CV	1.22 (1.17, 1.26)	< 0.001	1.22 (1.16, 1.28)	< 0.001	1.05 (1.04, 1.06)	< 0.001	1.03 (1.01, 1.04)	< 0.001
Quartiles of CV								
Q1	Reference		Reference		Reference		Reference	
Q2	1.32 (1.01, 1.72)	0.042	1.08 (0.82, 1.41)	0.596	1.35 (1.05, 1.73)	0.018	1.02 (0.79, 1.32)	0.853
Q3	1.87 (1.45, 2.40)	< 0.001	1.49 (1.16, 1.93)	0.002	1.46 (1.14, 1.86)	0.002	0.98 (0.76, 1.26)	0.892
Q4	2.71 (2.14, 3.45)	< 0.001	1.78 (1.39, 2.28)	< 0.001	2.22 (1.76, 2.79)	< 0.001	1.33 (1.04, 1.68)	0.022
**90‐day mortality**								
Continuous CV	1.19 (1.15, 1.23)	< 0.001	1.18 (1.13, 1.24)	< 0.001	1.04 (1.03, 1.05)	< 0.001	1.02 (1.01, 1.03)	< 0.001
Quartiles of CV								
Q1	Reference		Reference		Reference		Reference	
Q2	1.17 (0.94, 1.47)	0.158	0.98 (0.76, 1.20)	0.687	1.17 (0.94, 1.45)	0.161	0.89 (0.72, 1.11)	0.316
Q3	1.60 (1.30, 1.98)	< 0.001	1.31 (1.05, 1.62)	0.015	1.33 (1.08, 1.64)	0.008	0.90 (0.73, 1.13)	0.379
Q4	2.21 (1.81, 2.71)	< 0.001	1.51 (1.22, 1.87)	< 0.001	1.98 (1.63, 2.41)	< 0.001	1.23 (1.09, 1.51)	0.046
**360‐day mortality**								
Continuous CV	1.18 (1.14, 1.22)	< 0.001	1.17 (1.12, 1.22)	< 0.001	1.04 (1.03, 1.05)	< 0.001	1.49 (1.19, 1.87)	< 0.001
Quartiles of CV								
Q1	Reference		Reference		Reference		Reference	
Q2	1.20 (0.98, 1.47)	0.068	0.98 (0.80, 1.21)	0.885	1.23 (1.02, 1.49)	0.034	0.95 (0.78, 1.15)	0.594
Q3	1.57 (1.30, 1.90)	< 0.001	1.30 (1.07, 1.58)	0.008	1.31 (1.08, 1.58)	0.006	0.89 (0.74, 1.09)	0.284
Q4	2.13 (1.76, 2.56)	< 0.001	1.49 (1.23, 1.81)	< 0.001	1.92 (1.60, 2.30)	< 0.001	1.22 (1.02, 1.47)	0.045

*Note*: Sodium variability: Q1 < 0.94; 0.94 ≤ Q2 < 1.43; 1.43 ≤ Q3 < 2.04; Q4 ≥ 2.04. Potassium variability: Q1 < 5.25; 5.25 ≤ Q2 < 7.90; 7.90 ≤ Q3 < 11.62; Q4 ≥ 11.62. Model I: Unadjusted. Model II: Adjusted by age, male, race, heart rate, systolic blood pressure, diastolic blood pressure, respiratory rate, hypertensive, diabetes mellitus, chronic pulmonary disease, acute myocardial infarction, congestive heart failure, malignant tumor, chronic kidney disease, liver disease, platelets, hemoglobin, white blood cells, chloride, calcium, creatinine, glucose, Acute Physiology Score III, Glasgow Coma Scale, renal replacement therapy, mechanical ventilation, potassium supplementation, sodium supplementation, thrombolysis, antiplatelet drugs, warfarin, furosemide.

Abbreviations: CI, confidence interval; CV, coefficient of variation; HR, hazard ratio.

Similarly, for potassium variability (Table 2), continuous potassium variability showed a significant association with 30‐day (HR = 1.03, 95% CI: 1.01–1.04, *p* < 0.001), 90‐day (HR = 1.02, 95% CI: 1.01–1.03, *p* < 0.001), and 360‐day (HR = 1.49, 95% CI: 1.19–1.87, *p* < 0.001) mortality. For the quartiles of potassium variability, compared to Q1, Q4 was an independent risk factor for 30‐day (HR = 1.33, 95% CI: 1.04–1.68, *p* < 0.001), 90‐day (HR = 1.23, 95% CI: 1.09–1.51, *p* < 0.001), and 360‐day (HR = 1.22, 95% CI: 1.02–1.47, *p* < 0.001) mortality in Model II. Kaplan–Meier analyses revealed significant 30‐day, 90‐day, and 360‐day mortality differences across potassium variability, all log‐rank *p* < 0.001 (Figure 1). Patients in the Q4 exhibited significantly worse outcomes compared to Q1. RCS analyses (Figure 2) demonstrated a linear association between potassium variability and 30‐day (*p* for nonlinear = 0.435, *p* for overall < 0.001), 90‐day (*p* for nonlinear = 0.509, *p* for overall < 0.001), and 360‐day mortality (*p* for nonlinear = 0.627, *p* for overall < 0.001).

### 3.3 The Association Between Sodium and Potassium Variability and 30/90/360‐Day Mortality in NWICU

Table S7 presents the association between sodium and potassium variability and 30‐day, 90‐day, and 360‐day mortality. In Model II, for the quartiles of sodium variability, compared to Q1, Q4 was an independent risk factor for 30‐day (HR = 4.04, 95% CI: 2.13–7.63, *p* < 0.001), 90‐day (HR = 3.60, 95% CI: 2.00–6.48, *p* < 0.001), and 360‐day (HR = 3.21, 95% CI: 1.93–5.33, *p* < 0.001) mortality. However, continuous sodium variability showed no significant association with 30‐day, 90‐day, and 360‐day mortality. Kaplan–Meier analyses revealed significant 30‐day, 90‐day, and 360‐day mortality differences across sodium variability, all log‐rank *p* < 0.001 (Figure S2). Patients in the Q4 exhibited significantly worse outcomes compared to Q1. RCS analyses (Figure S3) demonstrated a nonlinear association between sodium variability and 30‐day (*p* for nonlinear < 0.001, *p* for overall < 0.001), 90‐day (*p* for nonlinear < 0.001, *p* for overall < 0.001), and 360‐day mortality (*p* for nonlinear < 0.001, *p* for overall < 0.001).

Similarly, for the quartiles of potassium variability (Table S7), compared to Q1, Q4 was an independent risk factor for 30‐day (HR = 2.04, 95% CI: 1.06–3.93, *p* = 0.033) mortality in Model II. However, Q4 of potassium variability showed no significant association with 90‐day and 360‐day mortality, and continuous potassium variation was not associated with ICU mortality. Kaplan–Meier analyses revealed significant 30‐day, 90‐day, and 360‐day mortality differences across potassium variability, all log‐rank *p* < 0.001 (Figure S2). RCS analyses (Figure S3) demonstrated a nonlinear association between potassium variability and 30‐day (*p* for nonlinear < 0.001, *p* for overall < 0.001), 90‐day (*p* for nonlinear = 0.002, *p* for overall = 0.002), and 360‐day mortality (*p* for nonlinear = 0.003, *p* for overall = 0.004).

### 3.4 The Association Between Combined Sodium–Potassium Variability and 30/90/360‐Day Mortality

Kaplan–Meier analyses revealed significant 30‐day, 90‐day, and 360‐day mortality differences across combined sodium–potassium variability, all log‐rank *p* < 0.001 (Figure S4).

**FIGURE 3:**
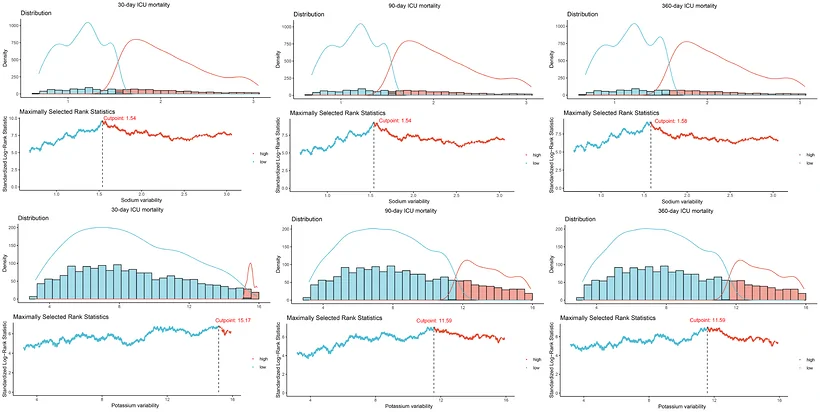
Determination of optimal risk stratification cut‐off points for sodium and potassium variability on 30‐day, 90‐day, and 360‐day mortality in the MIMIC‐IV.

Figure 3 and Figure S5 indicate that the optimal cut‐off values for sodium variability in 30‐day, 90‐day, and 360‐day mortality are 1.54, 1.54, and 1.58 respectively, whereas that for the optimal cut‐off values for potassium variability in 30‐day, 90‐day, and 360‐day mortality are 15.17, 11.59, and 11.59 in MIMIC‐IV respectively; the optimal cut‐off values for sodium variability in 30‐day, 90‐day, and 360‐day mortality are 1.97, whereas that for potassium variability in 30‐day, 90‐day, and 360‐day mortality are 11.78, 11.66, and 9.67 in NWICU respectively.

Based on these thresholds, patients were categorized into four groups according to low or high sodium and potassium variability: low sodium–low potassium variability (G1), low sodium–high potassium variability (G2), high sodium–low potassium variability (G3), and high sodium–high potassium variability (G4).

Table 3 and Table S8 present the association between combined sodium and potassium variability and 30‐day, 90‐day, and 360‐day mortality in MIMIC‐IV and NWICU. In Model II, compared to G1, G3 and G4 showed significant association with 30‐day (G3, HR = 1.53, 95% CI: 1.28–1.83, *p* < 0.001; G4, HR = 2.08, 95% CI: 1.61–2.68, *p* < 0.001), 90‐day (G3, HR = 1.46, 95% CI: 1.23–1.74, *p* < 0.001; G4, HR = 1.84, 95% CI: 1.51–2.23, *p* < 0.001), and 360‐day (G3, HR = 1.40, 95% CI: 1.20–1.64, *p* < 0.001; G4, HR = 1.73, 95% CI: 1.44–2.07, *p* < 0.001) mortality in MIMIC‐IV. Similarly, G3 and G4 showed significant association with 30‐day (G3, HR = 3.28, 95% CI: 1.97–5.45, *p* < 0.001; G4, HR = 5.42, 95% CI: 3.08–9.53, *p* < 0.001), 90‐day (G3, HR = 2.75, 95% CI: 1.75–4.33, *p* < 0.001; G4, HR = 4.32, 95% CI: 2.57–7.26, *p* < 0.001), and 360‐day (G3, HR = 3.34, 95% CI: 2.09–5.33, *p* < 0.001; G4, HR = 4.05, 95% CI: 2.54–6.47, *p* < 0.001) mortality in NWICU.

**TABLE 3:** The association between combined sodium–potassium variability and mortality in the MIMIC‐IV.

	Model I		Model II	
	HR (95% CI)	*p*	HR (95% CI)	*p*
**30‐day mortality**				
G1: Na^+^ CV < 1.54 and K^+^ CV < 15.17	Reference		Reference	
G2: Na^+^ CV < 1.54 and K^+^ CV > 15.17	1.64 (1.11, 2.42)	0.014	1.09 (0.73, 1.62)	0.691
G3: Na^+^ CV > 1.54 and K^+^ CV < 15.17	2.00 (1.68, 2.38)	< 0.001	1.53 (1.28, 1.83)	< 0.001
G4: Na^+^ CV > 1.54 and K^+^ CV > 15.17	3.51 (2.76, 4.45)	< 0.001	2.08 (1.61, 2.68)	< 0.001
**90‐day mortality**				
G1: Na^+^ CV < 1.54 and K^+^ CV < 11.59	Reference		Reference	
G2: Na^+^ CV < 1.54 and K^+^ CV > 11.59	1.52 (1.19, 1.94)	< 0.001	1.25 (0.97, 1.61)	0.081
G3: Na^+^ CV > 1.54 and K^+^ CV < 11.59	1.80 (1.52, 2.14)	< 0.001	1.46 (1.23, 1.74)	< 0.001
G4: Na^+^ CV > 1.54 and K^+^ CV > 11.59	2.74 (2.28, 3.30)	< 0.001	1.84 (1.51, 2.23)	< 0.001
**360‐day mortality**				
G1: Na^+^ CV < 1.58 and K^+^ CV < 11.59	Reference		Reference	
G2: Na^+^ CV < 1.58 and K^+^ CV > 11.59	1.49 (1.21, 1.85)	< 0.001	1.24 (0.99, 1.55)	0.052
G3: Na^+^ CV > 1.58 and K^+^ CV < 11.59	1.68 (1.45, 1.96)	< 0.001	1.40 (1.20, 1.64)	< 0.001
G4: Na^+^ CV > 1.58 and K^+^ CV > 11.59	2.48 (2.09, 2.95)	< 0.001	1.73 (1.44, 2.07)	< 0.001

*Note*: Model I: Unadjusted. Model II: Adjusted by age, male, race, heart rate, systolic blood pressure, diastolic blood pressure, respiratory rate, hypertensive, diabetes mellitus, chronic pulmonary disease, acute myocardial infarction, congestive heart failure, malignant tumor, chronic kidney disease, liver disease, platelets, hemoglobin, white blood cells, chloride, calcium, creatinine, glucose, Acute Physiology Score III, Glasgow Coma Scale, renal replacement therapy, mechanical ventilation, potassium supplementation, sodium supplementation, thrombolysis, antiplatelet drugs, warfarin, furosemide.

Abbreviations: CI, confidence interval; CV, coefficient of variation; HR, hazard ratio.

### 3.5 Sensitivity Analysis

To validate the robustness of our findings, several sensitivity analyses were conducted. First, using normal average electrolyte levels as references, we found that both dysnatremia (hypo‐ and hypernatremia) and hyperkalemia were significantly associated with increased 30‐, 90‐, and 360‐day mortality in the MIMIC‐IV cohort (Table S9). Similar trends were observed in the NWICU cohort for high sodium and potassium levels.

Second, we evaluated different clinical endpoints (ICU vs. in‐hospital mortality). In MIMIC‐IV, continuous variability and the highest quartile (Q4) of both sodium and potassium significantly increased the risk of in‐hospital and ICU mortality (Table S10). In NWICU, only Q4 sodium variability was significantly associated with increased in‐hospital mortality.

Third, after excluding patients with malignant tumors in the MIMIC‐IV cohort, both continuous variability and Q4 of sodium and potassium remained independently associated with increased mortality across all timeframes (Table S11).

Finally, to address potential residual confounding from complex interventions and metabolic derangements, after additionally adjusting for 24‐h net fluid balance, calculated osmolarity, glycemic control (glucose CV and insulin), glucose‐corrected sodium, sepsis, vasopressors, pH, and lactate, Q4 of sodium and potassium variability remained robustly associated with increased 30‐, 90‐, and 360‐day mortality (Table S12).

## 4 Discussion

This study demonstrated through two databases that fluctuations in sodium and potassium levels are significantly associated with both short‐term and long‐term mortality risks in ICU patients. Specifically, those in the highest quartile (Q4) for variability in sodium and potassium levels exhibited substantially higher mortality rates at 30, 90, and 360‐day post‐ICU admission. Furthermore, the combined analysis indicated that patients who experienced high variability in both sodium and potassium had the poorest prognosis, underlining the critical role of electrolyte stability in patient outcomes. These findings suggest that careful monitoring and management of electrolyte fluctuations could be vital in improving the survival and recovery of ICU patients, emphasizing the need for more targeted strategies to stabilize electrolyte levels in critically ill individuals.

Sodium and potassium fluctuations are critical in the pathophysiology of ischemic stroke. These electrolytes play pivotal roles in neuronal excitability, cellular osmoregulation, and vascular tone (Li et al. 2015). Variability in their levels can exacerbate brain injury through several mechanisms. Electrolyte imbalances can activate inflammatory pathways, leading to the release of pro‐inflammatory cytokines and the breakdown of the blood–brain barrier, facilitating edema and neuronal injury (Janssen et al. 2025; Chen et al. 2024). Studies have shown that early changes in blood gases and electrolytes occur in response to ischemic stroke, indicating their role in early brain injury (Zhu et al. 2022). Fluctuations in sodium and potassium levels can affect cerebral blood flow by altering vascular tone and impairing autoregulation, leading to ischemia or hemorrhage (Hu and Song 2017). Furthermore, variability in potassium levels can lead to cardiac arrhythmias, which are associated with poor outcomes in stroke patients (Crotti et al. 2020). Sodium concentration variability can serve as an independent predictor of mortality in critically ill patients, such as those with sepsis (Schrier et al. 2013). Harrois et al. (2021) demonstrated an independent association between daily serum sodium fluctuations and 28‐day mortality in traumatic brain injury patients (HR = 1.27, 95% CI: 1.01–1.61, *p* = 0.048), although their study did not specify a clear threshold for CV (Harrois et al. 2021). Our study further confirms the correlation between sodium variability and critically ill patients with ischemic stroke, particularly in relation to their long‐term ICU mortality, and defines the optimal threshold. This suggests that excessive fluctuations in serum sodium levels can disrupt the stability of hypothalamic osmoreceptor signaling, leading to an imbalance in vasopressin release, which exacerbates endothelial cell damage and triggers vasoconstriction or thrombus formation (Dmitrieva and Burg 2014).

A study involving 506 ICU patients found that higher variability in serum potassium levels was associated with an increased risk of 28‐day mortality. Specifically, patients with higher coefficients of variation in potassium levels had a significantly higher mortality rate compared to those with lower variability (Zhang et al. 2024). In a cohort of 5104 ICU patients with diabetes and sepsis, each 1 mmol/L increase in serum potassium was linked to a 25% higher risk of 28‐day mortality. Notably, hyperkalemia (> 5.0 mmol/L) was associated with an 86% higher risk compared to hypokalemia (< 3.5 mmol/L) (Cai et al. 2025). A study on maintenance hemodialysis patients found that higher serum potassium variability was independently associated with increased all‐cause and cardiovascular mortality, even within the normal potassium range (Men et al. 2024). Potassium plays a crucial role in maintaining cellular function and electrical stability in the heart and muscles (Palmer and Clegg 2019). Both hypokalemia and hyperkalemia can disrupt cardiac electrical activity, leading to arrhythmias, which are associated with increased mortality (Patel et al. 2017; Jensen et al. 2024). Potassium imbalances can impair neuromuscular function, leading to weakness and respiratory failure (Basok et al. 2021). Electrolyte disturbances can exacerbate systemic inflammation, contributing to organ dysfunction and mortality (Bouadma et al. 2019).

In this study, which analyzed 2593 patients from the MIMIC‐IV database and 673 patients from the NWICU cohort, we found that both sodium and potassium variability were independently associated with increased short‐ and long‐term mortality. In MIMIC‐IV, each one‐unit increase in sodium variability was associated with a 22% higher risk of 30‐day mortality (HR = 1.22, 95% CI: 1.16–1.28), while patients in the highest quartile (Q4) had a 1.78‐fold higher 30‐day mortality risk compared with those in Q1 (95% CI 1.39–2.28). Similarly, potassium variability in Q4 independently predicted 30‐day mortality (HR = 1.33, 95% CI: 1.04–1.68). When sodium and potassium fluctuations were combined, patients with high variability in both electrolytes (G4) showed markedly increased risks of death (30‐day HR = 2.08, 95% CI: 1.61–2.68), and this trend persisted at 90 and 360 days. These findings highlight that instability of electrolyte homeostasis, rather than absolute serum levels alone, is a critical determinant of outcome in critically ill patients. Crucially, the prognostic value of electrolyte variability in our study remained robust even after extensive adjustments for critical care interventions (e.g., fluid management, insulin therapy) and metabolic derangements (e.g., glycemic variability, acid–base status, calculated osmolarity). This suggests that while electrolyte fluctuations are undoubtedly influenced by therapeutic actions and acute illness severity, they are not mere bystanders. Instead, dynamic electrolyte instability represents an independent phenotype of high‐risk ischemic stroke patients, emphasizing the need for meticulous, continuous electrolyte monitoring. Regarding the discrepancy in continuous sodium variability results, the lack of significance in the NWICU cohort likely stems from its smaller sample size (*n* = 673) and limited statistical power. However, the Q4 of sodium variability consistently showed a strong, independent association with mortality in NWICU (30‐day mortality HR = 4.04, *p* < 0.001), aligning with the MIMIC‐IV findings. This suggests that while continuous metrics may be sensitive to sample size, extreme electrolyte instability remains a robust predictor of poor prognosis across different cohorts.

Our results expand upon previous studies that primarily focused on static dysnatremia or dyskalemia. Prior work demonstrated that both hypo‐ and hypernatremia independently predict mortality in hospitalized and ICU populations (Hu et al. 2017; Fan et al. 2022). Similarly, variability in potassium has been linked to poor outcomes in critical illness (Zhang et al. 2024; Hessels et al. 2015). This study has three significant aspects. First, we showed that fluctuations in sodium or potassium, even when mean levels remained within the normal range, were associated with elevated 30‐, 90‐, and 360‐day mortality. Second, these associations were consistent across two independent databases, multiple statistical models, and sensitivity analyses excluding patients with malignancy, indicating strong robustness. Third, we demonstrated a synergistic effect of combined sodium–potassium variability, where concurrent instability in both electrolytes conferred the highest mortality risk. Importantly, our VIF analysis demonstrated low collinearity between electrolyte variability and standard illness severity metrics like APS‐III. This indicates that dynamic electrolyte fluctuations are not merely surrogate markers for baseline disease severity; rather, they provide independent and incremental prognostic value. Monitoring these dynamic trends could thus complement traditional scoring systems in predicting ICU outcomes.

### 4.1 Limitations

This study has several limitations. First, it is retrospective, and despite extensive multivariable models and comprehensive sensitivity analyses addressing metabolic status and critical care interventions, we cannot completely rule out all unmeasured confounders inherent to observational database research, which may limit the accuracy of the results. Second, data from MIMIC‐IV and NWICU are from US hospitals, limiting generalizability, particularly in other regions, ethnic groups, and healthcare systems. Our population mainly includes ICU patients, which may not represent all ischemic stroke patients, especially those not requiring ICU admission. Third, generalized critical care databases such as MIMIC‐IV and NWICU lacked high‐resolution stroke‐specific prognostic variables. Key determinants of mortality, including National Institutes of Health Stroke Scale score, infarct volume, and lesion location, were not routinely available. Although reperfusion therapies such as thrombolysis were adjusted for when possible, limited neuroimaging detail remains an important limitation. Fourth, differences in baseline characteristics and adjustment variables between the two databases may affect the results. Fifth, ICU management is complex, and interactions between treatments may influence electrolyte fluctuations and mortality. Future prospective studies combining neurocritical care databases with high‐resolution stroke registries are needed to better isolate the impact of electrolyte variability from baseline neurological injury and to confirm this relationship.

## 5 Conclusions

This study demonstrates that variability in sodium and potassium levels is strongly associated with both short‐term and long‐term mortality among ICU patients. Patients with the greatest electrolyte fluctuations experienced substantially higher mortality at 30, 90, and 360 days. Notably, individuals with concurrent high sodium and potassium variability had the poorest prognosis, highlighting combined electrolyte instability as a particularly high‐risk phenotype. These findings underscore the clinical importance of monitoring and maintaining electrolyte stability in critically ill patients and suggest that reducing excessive electrolyte fluctuations may represent a modifiable target to improve ICU outcomes.

## Author Contributions

**Jianjun Zhu**: conceptualization, methodology, validation, Writing – review and editing, supervision. **Wei Zhang**: formal analysis, writing – review and editing. **Bin Zeng**: formal analysis, writing – review and editing. **Jian Zhang**: conceptualization, methodology, validation, writing – original draft. **Linfeng Xie**: data curation, formal analysis, writing – review and editing.

## Funding

This project was supported by the Scientific Research Project of Jiangsu Provincial Health Commission (K2023075).

## Ethics Statement

This study used de‐identified data from the publicly available MIMIC‐IV (v3.1) and NWICU (v0.1.0) databases. Ethical approval for the MIMIC‐IV database was obtained from the Institutional Review Boards of Beth Israel Deaconess Medical Center (2001‐P‐001699/14) and the Massachusetts Institute of Technology (0403000206). The NWICU database was approved by the Institutional Review Board of Northwestern University. As all data were de‐identified, additional ethical approval and informed consent were waived.

## Conflicts of Interest

The authors declare no conflicts of interest.

## Supporting information




**Supplementary Material**: brb371626‐sup‐0001‐SuppMat.docx

## Data Availability

The data used in this study are available to researchers who have approved access to the MIMIC‐IV and NWICU databases. Upon reasonable request and with proof of authorized access, the data supporting the findings of this study can be obtained from the corresponding author.
