# Supplementary material for: Dynamic Variability of Sodium and Potassium Predicts Mortality in Critically Ill Patients With Ischemic Stroke: Evidence From Two Critical Care Cohorts
Source: Brain Behav. 2026 Sep 4;16(9):e71626. doi: 10.1002/brb3.71626 (PMC13544567; doi:10.1002/brb3.71626)
Supplement: Supplementary file 1 [file BRB3-16-e71626-s001.docx]

Supplementary Table S1. Percentage of missing data of each variable in MIMIC-IV and NWICU.

Supplementary Table S2. Variance inflation factor of each variable in MIMIC-IV and NWICU.

Supplementary Table S3. Baseline characteristics classified by quartiles of sodium variability in the MIMIC-IV.

Supplementary Table S4. Baseline characteristics classified by quartiles of potassium variability in MIMIC-IV.

Supplementary Table S5. Baseline characteristics classified by quartiles of sodium variability in NWICU.

Supplementary Table S6. Baseline characteristics classified by quartiles of potassium variability in NWICU.

Supplementary Table S7. The associated between sodium and potassium variability and mortality in NWICU.

Supplementary Table S8. The associated between combined sodium-potassium variability and mortality in NWICU.

Supplementary Table S9. Sensitivity analysis of associated between mean sodium and potassium and 30/90/360-day mortality in MIMIC-IV and NWICU.

Supplementary Table S10. Sensitivity analysis of association between sodium and potassium variability and ICU/Hospital mortality in MIMIC-IV and NWICU.

Supplementary Table S11. Sensitivity analysis of association between sodium and potassium variability and mortality after excluding patients with tumours in MIMIC-IV.

Supplementary Table S12. Sensitivity analysis of association between sodium and potassium variability and mortality after adjusting other values in MIMIC-IV and NWICU.

Supplementary Figure S1. Flowchart of the study population.

Supplementary Figure S2. Kaplan-Meier survival curves between sodium and potassium variability and 30-day, 90-day, 360-day mortality in NWICU.

Supplementary Figure S3. The restricted cubic spline for the relationships between sodium and potassium variability and 30-day, 90-day, 360-day mortality in NWICU.

Supplementary Figure S4. Kaplan-Meier survival curves between combined sodium-potassium variability and 30-day, 90-day, 360-day mortality in MIMIC-IV (a, b, c) and NWICU (d, e, f).

Supplementary Figure S5. Determination of optimal risk stratification cut-off points for sodium and potassium variability on 30-day, 90-day, 360-day mortality in NWICU.

Supplementary Table S1. Percentage of missing data of each variable in MIMIC-IV and NWICU.

| **Characteristics** | **Missing count** | **Percentage, %** |
| --- | --- | --- |
| **MIMIC-IV** |  |  |
| Heart rate, bpm | 3/2,953 | 0.10% |
| SBP, mmHg | 3/2,953 | 0.10% |
| DBP, mmHg | 3/2,953 | 0.10% |
| Respiratory rate, bpm | 7/2,953 | 0.24% |
| Platelets, K/uL | 19/2,953 | 0.64% |
| Haemoglobin, g/dL | 19/2,953 | 0.64% |
| White blood cells, K/uL | 19/2,953 | 0.64% |
| Chloride, mmol/L | 10/2,953 | 0.34% |
| Calcium, mg/dL | 155/2,953 | 5.25% |
| Initial sodium, mEq/L | 10/2,953 | 0.34% |
| Initial potassium, mEq/L | 10/2,953 | 0.34% |
| Creatinine, mg/dL | 3/2,953 | 0.10% |
| Glucose, mg/dL | 11/2,953 | 0.37% |
| GCS score | 4/2,953 | 0.14% |
|  |  |  |
| **NWICU** |  |  |
| Heart rate, bpm | 88/673 | 13.07% |
| SBP, mmHg | 91/673 | 13.52% |
| DBP, mmHg | 89/673 | 13.22% |
| Respiratory rate, bpm | 80/673 | 11.88% |
| Platelets, K/uL | 35/673 | 5.20% |
| Haemoglobin, g/dL | 48/673 | 7.13% |
| White blood cells, K/uL | 38/673 | 5.64% |
| Chloride, mmol/L | 16/673 | 2.38% |
| Calcium, mg/dL | 17/673 | 2.53% |
| Initial sodium, mEq/L | 14/673 | 2.08% |
| Initial potassium, mEq/L | 16/673 | 2.38% |
| Creatinine, mg/dL | 17/673 | 2.53% |
| Glucose, mg/dL | 10/673 | 1.49% |

Abbreviations: SBP, systolic blood pressure; DBP, diastolic blood pressure; GCS score: Glasgow Coma Scale score.

Supplementary Table S2. Variance inflation factor of each variable in MIMIC-IV and NWICU.

| **Characteristics** | **MIMIC-IV** | **NWICU** |
| --- | --- | --- |
| Age | 1.441 | 1.106 |
| Male | 1.178 | 1.113 |
| Race | 1.048 | 1.132 |
| Heart rate | 1.488 | 1.334 |
| Systolic blood pressure | 1.806 | 2.127 |
| Diastolic blood pressure | 2.218 | 2.075 |
| Respiratory rate | 1.306 | 1.193 |
| Hypertensive | 1.485 | 1.253 |
| Diabetes mellitus | 1.294 | 1.335 |
| Chronic pulmonary disease | 1.043 | 1.095 |
| Acute myocardial infarction | 1.151 | 1.17 |
| Congestive heart failure | 1.31 | 1.294 |
| Malignant tumor | 1.088 | 1.248 |
| Chronic kidney disease | 1.592 | 1.111 |
| Liver disease | 1.105 | 1.239 |
| Platelets | 1.247 | 1.452 |
| Haemoglobin | 1.629 | 1.375 |
| White blood cells | 1.338 | 3.852 |
| Chloride | 2.52 | 1.526 |
| Calcium | 1.502 | 2.617 |
| Initial sodium | 3.297 | 1.291 |
| Initial potassium | 2.278 | 8.572 |
| Mean sodium | 2.307 | 1.795 |
| Mean potassium | 2.326 | 2.898 |
| Creatinine | 1.742 | 1.387 |
| Glucose | 1.387 | 1.137 |
| Acute Physiology Score III | 2.13 | - |
| Glasgow Coma Scale score | 1.324 | - |
| Renal Replacement Therapy | 1.154 | 1.228 |
| Invasive Mechanical ventilation | 1.516 | 1.201 |
| Potassium supplementation | 1.305 | 1.168 |
| Sodium supplementation | 1.09 | 1.123 |
| Thrombolysis | 1.182 | - |
| Antiplatelet drugs | 1.027 | 1.056 |
| Warfarin | 1.119 | 1.039 |
| Furosemide | 1.041 | 1.185 |
| Sodium variability | 1.173 | 2.322 |
| Potassium variability | 1.304 | 1.998 |

A variance inflation factor of <5 for each variable suggested the absence of multicollinearity

Supplementary Table S3. Baseline characteristics classified by quartiles of sodium variability in the MIMIC-IV.

| **Characteristics** | **ALL**  **(n = 2,593)** | **Q1**  **(n = 615)** | **Q2**  **(n = 649)** | **Q3**  **(n = 667)** | **Q4**  **(n = 662)** | ***P value*** |
| --- | --- | --- | --- | --- | --- | --- |
| Age, years | 69.0 (59.0, 80.0) | 71.0 (59.0, 80.0) | 70.0 (59.0, 80.0) | 70.0 (58.0, 80.0) | 68.0 (58.0, 78.0) | 0.079 |
| Male, n (%) | 1321 (50.9) | 343 (55.8) | 328 (50.5) | 308 (46.2) | 342 (51.7) | 0.007 |
| Race, n (%) |  |  |  |  |  | 0.074 |
| White | 1497 (57.7) | 360 (58.5) | 390 (60.1) | 399 (59.8) | 348 (52.6) |  |
| Black | 225 ( 8.7) | 42 (6.8) | 57 (8.8) | 54 (8.1) | 72 (10.9) |  |
| Hispanic | 76 ( 2.9) | 17 (2.8) | 21 (3.2) | 20 (3) | 18 (2.7) |  |
| Asian | 78 ( 3.0) | 20 (3.3) | 17 (2.6) | 25 (3.7) | 16 (2.4) |  |
| Others | 717 (27.7) | 176 (28.6) | 164 (25.3) | 169 (25.3) | 208 (31.4) |  |
| Vital signs |  |  |  |  |  |  |
| Heart rate, bpm | 80.4 (70.6, 91.6) | 77.3 (68.2, 86.3) | 81.1 (70.9, 92.4) | 81.1 (71.0, 92.8) | 82.4 (71.9, 93.4) | < 0.001 |
| SBP, mmHg | 128.5 (115.9, 142.6) | 131.0 (118.1, 144.2) | 126.6 (114.6, 141.0) | 128.0 (115.7, 142.6) | 128.4 (116.0, 141.1) | 0.010 |
| DBP, mmHg | 67.5 (59.7, 77.2) | 70.0 (61.9, 79.5) | 66.8 (58.5, 77.2) | 67.3 (59.1, 75.6) | 66.1 (59.6, 76.5) | < 0.001 |
| Respiratory rate, bpm | 18.8 (16.9, 21.4) | 18.4 (16.8, 20.3) | 18.8 (16.9, 21.4) | 19.0 (16.9, 21.6) | 19.1 (17.1, 21.9) | < 0.001 |
| Comorbidities, n (%) |  |  |  |  |  |  |
| Hypertensive | 1331 (51.3) | 329 (53.5) | 331 (51) | 336 (50.4) | 335 (50.6) | 0.667 |
| Diabetes mellitus | 563 (21.7) | 107 (17.4) | 139 (21.4) | 146 (21.9) | 171 (25.8) | 0.004 |
| Chronic pulmonary disease | 157 ( 6.1) | 37 (6) | 47 (7.2) | 38 (5.7) | 35 (5.3) | 0.485 |
| Acute myocardial infarction | 254 ( 9.8) | 39 (6.3) | 68 (10.5) | 80 (12) | 67 (10.1) | 0.006 |
| Congestive heart failure | 628 (24.2) | 116 (18.9) | 176 (27.1) | 165 (24.7) | 171 (25.8) | 0.003 |
| Malignant tumor | 247 ( 9.5) | 59 (9.6) | 60 (9.2) | 69 (10.3) | 59 (8.9) | 0.832 |
| Chronic kidney disease | 432 (16.7) | 101 (16.4) | 108 (16.6) | 105 (15.7) | 118 (17.8) | 0.783 |
| Liver disease | 105 ( 4.0) | 19 (3.1) | 22 (3.4) | 27 (4) | 37 (5.6) | 0.101 |
| Laboratory tests |  |  |  |  |  |  |
| Platelets, K/uL | 207.0 (157.0, 270.0) | 204.0 (161.0, 262.5) | 208.0 (157.0, 267.0) | 210.0 (160.5, 280.0) | 207.0 (148.2, 270.0) | 0.223 |
| Haemoglobin, g/dL | 11.6 (9.8, 13.3) | 11.9 (10.3, 13.4) | 11.6 (9.7, 13.3) | 11.5 (9.8, 13.1) | 11.5 (9.5, 13.3) | 0.026 |
| White blood cells, K/uL | 10.6 (8.0, 14.2) | 9.7 (7.7, 12.6) | 10.6 (7.6, 14.2) | 10.5 (8.0, 14.3) | 11.7 (8.6, 15.2) | < 0.001 |
| Chloride, mmol/L | 104.0 (101.0, 107.0) | 105.0 (102.0, 107.0) | 105.0 (101.0, 108.0) | 104.0 (100.0, 107.0) | 104.0 (100.0, 108.0) | 0.002 |
| Calcium, mg/dL | 8.6 (8.1, 9.0) | 8.7 (8.2, 9.1) | 8.6 (8.1, 9.0) | 8.6 (8.1, 9.0) | 8.6 (8.0, 9.0) | 0.011 |
| Initial sodium, mEq/L | 139.0 (136.0, 142.0) | 140.0 (138.0, 142.0) | 139.0 (137.0, 142.0) | 139.0 (136.0, 141.0) | 139.0 (135.0, 142.0) | < 0.001 |
| Initial potassium, mEq/L | 4.1 (3.7, 4.5) | 4.1 (3.8, 4.4) | 4.1 (3.8, 4.5) | 4.1 (3.7, 4.5) | 4.0 (3.6, 4.5) | 0.419 |
| Mean sodium, mEq/L | 139.8 (137.3, 142.3) | 139.7 (137.7, 141.6) | 139.7 (137.2, 141.8) | 139.2 (137.0, 141.6) | 141.1 (137.4, 145.6) | < 0.001 |
| Mean potassium, mEq/L | 4.0 (3.8, 4.3) | 4.0 (3.8, 4.2) | 4.0 (3.8, 4.3) | 4.0 (3.8, 4.3) | 4.0 (3.7, 4.3) | 0.048 |
| Creatinine, mg/dL | 0.9 (0.7, 1.3) | 0.9 (0.7, 1.1) | 0.9 (0.7, 1.2) | 0.9 (0.7, 1.3) | 1.0 (0.7, 1.4) | < 0.001 |
| Glucose, mg/dL | 128.0 (104.0, 165.0) | 118.0 (100.0, 146.0) | 125.0 (103.0, 163.0) | 132.0 (108.0, 169.0) | 135.0 (109.0, 181.0) | < 0.001 |
| Clinical scoring |  |  |  |  |  |  |
| APS-III | 39.0 (29.0, 53.0) | 34.0 (26.0, 47.0) | 40.0 (31.0, 53.0) | 40.0 (31.0, 54.0) | 43.0 (31.0, 59.0) | < 0.001 |
| GCS score | 15.0 (14.0, 15.0) | 15.0 (14.0, 15.0) | 15.0 (14.0, 15.0) | 15.0 (13.0, 15.0) | 15.0 (14.0, 15.0) | 0.217 |
| Intervention, n (%) |  |  |  |  |  |  |
| Renal Replacement Therapy | 43 ( 1.7) | 3 (0.5) | 9 (1.4) | 16 (2.4) | 15 (2.3) | 0.027 |
| Invasive Mechanical ventilation | 1160 (44.7) | 201 (32.7) | 271 (41.8) | 293 (43.9) | 395 (59.7) | < 0.001 |
| Potassium supplementation | 610 (23.5) | 107 (17.4) | 150 (23.1) | 166 (24.9) | 187 (28.2) | < 0.001 |
| Sodium supplementation | 1769 (68.2) | 412 (67) | 438 (67.5) | 457 (68.5) | 462 (69.8) | 0.713 |
| Thrombolysis | 53 ( 2.0) | 12 (2) | 15 (2.3) | 14 (2.1) | 12 (1.8) | 0.931 |
| Antiplatelet drugs | 925 (35.7) | 213 (34.6) | 243 (37.4) | 259 (38.8) | 210 (31.7) | 0.035 |
| Warfarin | 153 ( 5.9) | 43 (7) | 41 (6.3) | 37 (5.5) | 32 (4.8) | 0.387 |
| Furosemide | 166 ( 6.4) | 21 (3.4) | 25 (3.9) | 51 (7.6) | 69 (10.4) | < 0.001 |
| Clinical Outcomes |  |  |  |  |  |  |
| 30-day mortality, n (%) | 638 (24.6) | 94 (15.3) | 128 (19.7) | 178 (26.7) | 238 (36) | < 0.001 |
| 90-day mortality, n (%) | 812 (31.3) | 140 (22.8) | 169 (26) | 223 (33.4) | 280 (42.3) | < 0.001 |
| 360-day mortality, n (%) | 976 (37.6) | 173 (28.1) | 213 (32.8) | 266 (39.9) | 324 (48.9) | < 0.001 |
| ICU length of stay, day | 4.2 (2.2, 8.0) | 3.3 (2.0, 6.1) | 4.2 (2.3, 8.0) | 4.2 (2.2, 8.2) | 5.0 (2.6, 9.9) | < 0.001 |
| Length of hospital stay, day | 9.9 (5.8, 17.8) | 8.1 (4.8, 15.4) | 9.8 (6.1, 17.9) | 10.5 (6.3, 18.1) | 11.9 (6.1, 20.7) | < 0.001 |

Abbreviations: APS-III: Acute Physiology Score III; DBP, diastolic blood pressure; GCS score: Glasgow Coma Scale score; MIMIC-IV, Medical Information Mart for Intensive Care IV; SBP, systolic blood pressure.

Supplementary Table S4. Baseline characteristics classified by quartiles of potassium variability in MIMIC-IV.

| **Characteristics** | **ALL**  **(n = 2,593)** | **Q1**  **(n=624)** | **Q2**  **(n=661)** | **Q3**  **(n=654)** | **Q4**  **(n=654)** | ***P value*** |
| --- | --- | --- | --- | --- | --- | --- |
| Age, years | 69.0 (59.0, 80.0) | 68.0 (57.0, 80.0) | 69.0 (59.0, 79.0) | 70.0 (59.0, 80.0) | 70.0 (59.0, 80.0) | 0.851 |
| Male, n (%) | 1321 (50.9) | 344 (55.1) | 374 (56.6) | 307 (46.9) | 296 (45.3) | < 0.001 |
| Race, n (%) |  |  |  |  |  | 0.337 |
| White | 1497 (57.7) | 367 (58.8) | 370 (56) | 384 (58.7) | 376 (57.5) |  |
| Black | 225 ( 8.7) | 56 (9) | 50 (7.6) | 57 (8.7) | 62 (9.5) |  |
| Hispanic | 76 ( 2.9) | 24 (3.8) | 21 (3.2) | 12 (1.8) | 19 (2.9) |  |
| Asian | 78 ( 3.0) | 22 (3.5) | 24 (3.6) | 13 (2) | 19 (2.9) |  |
| Others | 717 (27.7) | 155 (24.8) | 196 (29.7) | 188 (28.7) | 178 (27.2) |  |
| Vital signs |  |  |  |  |  |  |
| Heart rate, bpm | 80.4 (70.6, 91.6) | 78.5 (67.7, 89.5) | 79.1 (70.7, 90.3) | 82.2 (71.6, 93.4) | 81.8 (71.9, 93.1) | < 0.001 |
| SBP, mmHg | 128.5 (115.9, 142.6) | 132.7 (118.6, 146.7) | 128.8 (116.7, 140.9) | 127.8 (115.2, 142.4) | 124.8 (112.8, 139.1) | < 0.001 |
| DBP, mmHg | 67.5 (59.7, 77.2) | 70.1 (61.6, 79.8) | 67.5 (58.9, 76.8) | 66.8 (58.9, 75.8) | 66.5 (59.0, 75.6) | < 0.001 |
| Respiratory rate, bpm | 18.8 (16.9, 21.4) | 18.3 (16.7, 20.3) | 18.7 (16.7, 21.2) | 19.1 (17.2, 21.8) | 19.2 (17.2, 21.8) | < 0.001 |
| Comorbidities, n (%) |  |  |  |  |  |  |
| Hypertensive | 1331 (51.3) | 322 (51.6) | 354 (53.6) | 332 (50.8) | 323 (49.4) | 0.494 |
| Diabetes mellitus | 563 (21.7) | 144 (23.1) | 120 (18.2) | 142 (21.7) | 157 (24) | 0.054 |
| Chronic pulmonary disease | 157 ( 6.1) | 30 (4.8) | 49 (7.4) | 38 (5.8) | 40 (6.1) | 0.27 |
| Acute myocardial infarction | 254 ( 9.8) | 44 (7.1) | 52 (7.9) | 85 (13) | 73 (11.2) | < 0.001 |
| Congestive heart failure | 628 (24.2) | 124 (19.9) | 158 (23.9) | 175 (26.8) | 171 (26.1) | 0.018 |
| Malignant tumor | 247 ( 9.5) | 54 (8.7) | 65 (9.8) | 64 (9.8) | 64 (9.8) | 0.867 |
| Chronic kidney disease | 432 (16.7) | 103 (16.5) | 104 (15.7) | 107 (16.4) | 118 (18) | 0.714 |
| Liver disease | 105 ( 4.0) | 16 (2.6) | 25 (3.8) | 34 (5.2) | 30 (4.6) | 0.095 |
| Laboratory tests |  |  |  |  |  |  |
| Platelets, K/uL | 207.0 (157.0, 270.0) | 207.0 (163.5, 265.0) | 207.0 (152.0, 266.0) | 211.0 (160.0, 273.0) | 203.5 (152.0, 274.8) | 0.528 |
| Haemoglobin, g/dL | 11.6 (9.8, 13.3) | 12.0 (10.3, 13.7) | 11.6 (9.6, 13.2) | 11.4 (9.4, 13.0) | 11.5 (9.7, 13.2) | < 0.001 |
| White blood cells, K/uL | 10.6 (8.0, 14.2) | 9.7 (7.7, 12.5) | 10.7 (8.2, 14.2) | 10.7 (7.7, 14.3) | 11.8 (8.6, 15.5) | < 0.001 |
| Chloride, mmol/L | 104.0 (101.0, 107.0) | 104.0 (102.0, 107.0) | 105.0 (101.0, 108.0) | 104.0 (101.0, 108.0) | 104.0 (100.0, 107.0) | 0.057 |
| Calcium, mg/dL | 8.6 (8.1, 9.0) | 8.7 (8.3, 9.1) | 8.6 (8.1, 9.0) | 8.6 (8.1, 9.0) | 8.5 (7.9, 8.9) | < 0.001 |
| Initial sodium, mEq/L | 139.0 (136.0, 142.0) | 139.0 (137.0, 142.0) | 139.0 (136.0, 141.0) | 139.0 (137.0, 142.0) | 139.0 (136.0, 141.0) | < 0.001 |
| Initial potassium, mEq/L | 4.1 (3.7, 4.5) | 4.0 (3.8, 4.2) | 4.1 (3.8, 4.4) | 4.1 (3.7, 4.5) | 4.2 (3.5, 5.0) | < 0.001 |
| Mean sodium, mEq/L | 139.8 (137.3, 142.3) | 140.1 (138.0, 142.0) | 139.8 (137.0, 142.2) | 139.7 (137.2, 142.5) | 139.3 (136.8, 142.5) | 0.116 |
| Mean potassium, mEq/L | 4.0 (3.8, 4.3) | 4.0 (3.8, 4.2) | 4.0 (3.8, 4.3) | 3.9 (3.7, 4.2) | 4.1 (3.8, 4.4) | < 0.001 |
| Creatinine, mg/dL | 0.9 (0.7, 1.3) | 0.9 (0.7, 1.1) | 0.9 (0.7, 1.2) | 0.9 (0.7, 1.2) | 1.0 (0.7, 1.4) | 0.004 |
| Glucose, mg/dL | 128.0 (104.0, 165.0) | 120.0 (102.0, 150.2) | 126.0 (105.0, 157.0) | 128.0 (105.0, 169.8) | 135.0 (108.0, 183.8) | < 0.001 |
| Clinical scoring |  |  |  |  |  |  |
| APS-III | 39.0 (29.0, 53.0) | 35.0 (26.0, 47.0) | 38.0 (29.0, 52.0) | 41.0 (30.0, 53.0) | 44.0 (32.0, 60.0) | < 0.001 |
| GCS score | 15.0 (14.0, 15.0) | 15.0 (13.0, 15.0) | 15.0 (14.0, 15.0) | 15.0 (14.0, 15.0) | 15.0 (14.0, 15.0) | 0.031 |
| Intervention, n (%) |  |  |  |  |  |  |
| Renal Replacement Therapy | 43 ( 1.7) | 7 (1.1) | 9 (1.4) | 5 (0.8) | 22 (3.4) | < 0.001 |
| Mechanical ventilation | 1160 (44.7) | 167 (26.8) | 299 (45.2) | 317 (48.5) | 377 (57.6) | < 0.001 |
| Potassium supplementation | 610 (23.5) | 103 (16.5) | 149 (22.5) | 176 (26.9) | 182 (27.8) | < 0.001 |
| Sodium supplementation | 1769 (68.2) | 435 (69.7) | 447 (67.6) | 430 (65.7) | 457 (69.9) | 0.331 |
| Thrombolysis | 53 ( 2.0) | 12 (1.9) | 17 (2.6) | 7 (1.1) | 17 (2.6) | 0.167 |
| Antiplatelet drugs | 925 (35.7) | 235 (37.7) | 237 (35.9) | 237 (36.2) | 216 (33) | 0.366 |
| Warfarin | 153 ( 5.9) | 43 (6.9) | 45 (6.8) | 36 (5.5) | 29 (4.4) | 0.187 |
| Furosemide | 166 ( 6.4) | 19 (3) | 37 (5.6) | 48 (7.3) | 62 (9.5) | < 0.001 |
| Clinical Outcomes |  |  |  |  |  |  |
| 30-day mortality, n (%) | 638 (24.6) | 108 (17.3) | 149 (22.5) | 158 (24.2) | 223 (34.1) | < 0.001 |
| 90-day mortality, n (%) | 812 (31.3) | 153 (24.5) | 182 (27.5) | 202 (30.9) | 275 (42) | < 0.001 |
| 360-day mortality, n (%) | 976 (37.6) | 186 (29.8) | 233 (35.2) | 240 (36.7) | 317 (48.5) | < 0.001 |
| ICU length of stay, day | 4.2 (2.2, 8.0) | 3.7 (2.0, 6.6) | 4.2 (2.2, 8.5) | 4.4 (2.4, 8.5) | 4.7 (2.4, 8.5) | < 0.001 |
| Length of hospital stay, day | 9.9 (5.8, 17.8) | 8.0 (5.0, 15.4) | 10.2 (5.9, 18.1) | 11.2 (6.4, 19.9) | 10.1 (6.1, 18.5) | < 0.001 |

Abbreviations: SBP, systolic blood pressure; DBP, diastolic blood pressure; APS-III: Acute Physiology Score III; GCS score: Glasgow Coma Scale score.

Supplementary Table S5. Baseline characteristics classified by quartiles of sodium variability in NWICU.

| **Characteristics** | **ALL**  **(n = 673)** | **Q1**  **(n=169)** | **Q2**  **(n=169)** | **Q3**  **(n=167)** | **Q4**  **(n=168)** | ***P value*** |
| --- | --- | --- | --- | --- | --- | --- |
| Age, years | 69.0 (60.0, 78.0) | 71.0 (61.0, 80.0) | 69.0 (61.0, 78.0) | 70.0 (60.0, 78.0) | 67.0 (58.0, 77.0) | 0.070 |
| Male, n (%) | 370 (55.0) | 87 (51.5) | 96 (56.8) | 95 (56.9) | 92 (54.8) | 0.726 |
| Race, n (%) |  |  |  |  |  | 0.669 |
| White | 433 (64.3) | 108 (63.9) | 118 (69.8) | 108 (64.7) | 99 (58.9) |  |
| Black | 104 (15.5) | 26 (15.4) | 21 (12.4) | 27 (16.2) | 30 (17.9) |  |
| Asian | 2 ( 0.3) | 0 (0) | 0 (0) | 1 (0.6) | 1 (0.6) |  |
| Others | 134 (19.9) | 35 (20.7) | 30 (17.8) | 31 (18.6) | 38 (22.6) |  |
| Vital signs |  |  |  |  |  |  |
| Heart rate, bpm | 84.0 (73.0, 98.0) | 82.0 (74.0, 98.0) | 80.0 (71.0, 92.8) | 87.5 (72.2, 99.8) | 87.0 (77.0, 103.0) | 0.021 |
| SBP, mmHg | 138.0 (119.0, 160.0) | 140.5 (123.0, 162.0) | 135.5 (117.2, 168.5) | 137.5 (116.2, 160.8) | 137.5 (115.2, 154.5) | 0.476 |
| DBP, mmHg | 74.0 (63.0, 87.0) | 76.0 (64.0, 87.0) | 73.0 (63.0, 88.0) | 74.0 (65.0, 86.0) | 74.0 (62.0, 86.5) | 0.983 |
| Respiratory rate, bpm | 20.0 (16.0, 24.0) | 20.0 (16.0, 24.0) | 19.0 (16.0, 22.0) | 19.0 (16.0, 23.0) | 21.0 (17.0, 26.0) | 0.197 |
| Comorbidities, n (%) |  |  |  |  |  |  |
| Hypertensive | 145 (21.5) | 36 (21.3) | 35 (20.7) | 38 (22.8) | 36 (21.4) | 0.974 |
| Diabetes mellitus | 89 (13.2) | 19 (11.2) | 23 (13.6) | 24 (14.4) | 23 (13.7) | 0.844 |
| Chronic pulmonary disease | 18 ( 2.7) | 1 (0.6) | 9 (5.3) | 2 (1.2) | 6 (3.6) | 0.027 |
| Acute myocardial infarction | 28 ( 4.2) | 4 (2.4) | 4 (2.4) | 8 (4.8) | 12 (7.1) | 0.084 |
| Congestive heart failure | 40 ( 5.9) | 9 (5.3) | 11 (6.5) | 8 (4.8) | 12 (7.1) | 0.791 |
| Malignant tumor | 0 (0.0) | 0 (0.0) | 0 (0.0) | 0 (0.0) | 0 (0.0) | - |
| Chronic kidney disease | 40 ( 5.9) | 13 (7.7) | 8 (4.7) | 9 (5.4) | 10 (6) | 0.692 |
| Liver disease | 17 ( 2.5) | 2 (1.2) | 5 (3) | 4 (2.4) | 6 (3.6) | 0.564 |
| Laboratory tests |  |  |  |  |  |  |
| Platelets, K/uL | 209.0 (161.0, 268.8) | 209.0 (174.5, 262.5) | 208.0 (163.0, 262.5) | 206.0 (161.8, 273.0) | 217.0 (148.8, 278.2) | 0.976 |
| Haemoglobin, g/dL | 12.1 (10.0, 13.6) | 12.3 (10.3, 13.8) | 12.6 (10.3, 13.8) | 11.6 (9.8, 13.5) | 11.8 (9.3, 13.5) | 0.090 |
| White blood cells, K/uL | 10.2 (7.8, 13.9) | 8.8 (7.2, 11.8) | 10.2 (7.7, 13.8) | 10.1 (8.0, 14.4) | 11.6 (8.6, 15.3) | < 0.001 |
| Chloride, mmol/L | 106.0 (102.0, 109.0) | 106.0 (103.0, 108.0) | 106.0 (103.0, 108.0) | 107.0 (103.0, 109.0) | 105.0 (100.0, 108.0) | 0.034 |
| Calcium, mg/dL | 8.6 (8.2, 9.0) | 8.7 (8.3, 9.0) | 8.7 (8.2, 9.1) | 8.5 (8.1, 9.0) | 8.4 (7.9, 8.9) | 0.033 |
| Initial sodium, mEq/L | 139.0 (136.0, 141.0) | 139.0 (137.0, 141.0) | 139.0 (137.0, 141.0) | 140.0 (136.0, 142.0) | 138.0 (134.0, 142.0) | 0.137 |
| Initial potassium, mEq/L | 3.9 (3.6, 4.2) | 3.9 (3.6, 4.2) | 3.9 (3.6, 4.2) | 3.9 (3.5, 4.2) | 3.9 (3.6, 4.3) | 0.627 |
| Mean sodium, mEq/L | 139.8 (137.4, 142.3) | 139.0 (137.3, 141.0) | 139.3 (137.0, 141.0) | 139.6 (137.4, 142.5) | 142.6 (138.7, 148.6) | < 0.001 |
| Mean potassium, mEq/L | 3.9 (3.7, 4.1) | 3.9 (3.7, 4.1) | 3.9 (3.7, 4.1) | 3.9 (3.7, 4.2) | 3.9 (3.7, 4.1) | 0.968 |
| Creatinine, mg/dL | 106.0 (102.0, 109.0) | 106.0 (103.0, 108.0) | 106.0 (103.0, 109.0) | 107.0 (103.0, 109.0) | 105.0 (100.0, 108.0) | 0.030 |
| Glucose, mg/dL | 134.0 (110.0, 174.5) | 124.0 (104.2, 154.8) | 131.0 (106.0, 174.0) | 142.0 (113.0, 187.0) | 139.5 (119.8, 206.0) | < 0.001 |
| Intervention, n (%) |  |  |  |  |  |  |
| Haemodialysis | 7 ( 1.0) | 3 (1.8) | 1 (0.6) | 2 (1.2) | 1 (0.6) | 0.755 |
| IMV | 150 (22.3) | 19 (11.2) | 33 (19.5) | 38 (22.8) | 60 (35.7) | < 0.001 |
| Potassium supplementation | 140 (20.8) | 37 (21.9) | 37 (21.9) | 29 (17.4) | 37 (22) | 0.661 |
| Sodium supplementation | 294 (43.7) | 63 (37.3) | 73 (43.2) | 76 (45.5) | 82 (48.8) | 0.183 |
| Antiplatelet drugs | 227 (33.7) | 71 (42) | 62 (36.7) | 44 (26.3) | 50 (29.8) | 0.011 |
| Warfarin | 6 ( 0.9) | 2 (1.2) | 3 (1.8) | 1 (0.6) | 0 (0) | 0.498 |
| Furosemide | 48 ( 7.1) | 10 (5.9) | 11 (6.5) | 12 (7.2) | 15 (8.9) | 0.730 |
| Clinical Outcomes |  |  |  |  |  |  |
| 30-day mortality, n (%) | 109 (16.2) | 13 (7.7) | 13 (7.7) | 26 (15.6) | 57 (33.9) | < 0.001 |
| 90-day mortality, n (%) | 128 (19.0) | 16 (9.5) | 21 (12.4) | 30 (18) | 61 (36.3) | < 0.001 |
| 360-day mortality, n (%) | 151 (22.4) | 23 (13.6) | 25 (14.8) | 35 (21) | 68 (40.5) | < 0.001 |
| ICU length of stay, day | 4.0 (2.8, 6.8) | 3.0 (2.2, 4.1) | 3.7 (2.7, 5.2) | 4.7 (2.9, 7.3) | 7.0 (4.6, 12.9) | < 0.001 |
| Length of hospital stay, day | 8.8 (5.2, 15.9) | 6.6 (4.0, 10.2) | 7.1 (4.9, 13.5) | 9.6 (6.1, 15.3) | 13.7 (7.3, 21.7) | < 0.001 |

Abbreviations: SBP, systolic blood pressure; DBP, diastolic blood pressure; IMV, IMV, Invasive mechanical ventilation.

Supplementary Table S6. Baseline characteristics classified by quartiles of potassium variability in NWICU.

| **Characteristics** | **ALL**  **(n = 673)** | **Q1**  **(n=169)** | **Q2**  **(n=168)** | **Q3**  **(n=168)** | **Q4**  **(n=168)** | ***P value*** |
| --- | --- | --- | --- | --- | --- | --- |
| Age, years | 69.0 (60.0, 78.0) | 70.0 (63.0, 79.0) | 69.0 (58.0, 78.0) | 69.0 (60.0, 77.0) | 68.0 (58.0, 76.2) | 0.206 |
| Male, n (%) | 370 (55.0) | 106 (62.7) | 103 (61.3) | 88 (52.4) | 73 (43.5) | < 0.001 |
| Race, n (%) |  |  |  |  |  | 0.550 |
| White | 433 (64.3) | 113 (66.9) | 112 (66.7) | 105 (62.5) | 103 (61.3) |  |
| Black | 104 (15.5) | 25 (14.8) | 25 (14.9) | 22 (13.1) | 32 (19) |  |
| Asian | 2 ( 0.3) | 0 (0) | 0 (0) | 2 (1.2) | 0 (0) |  |
| Others | 134 (19.9) | 31 (18.3) | 31 (18.5) | 39 (23.2) | 33 (19.6) |  |
| Vital signs |  |  |  |  |  |  |
| Heart rate, bpm | 84.0 (73.0, 98.0) | 79.0 (70.0, 91.0) | 86.0 (74.0, 97.0) | 85.0 (74.2, 102.0) | 87.0 (75.2, 99.8) | 0.014 |
| SBP, mmHg | 138.0 (119.0, 160.0) | 146.0 (123.0, 168.0) | 137.0 (121.8, 154.2) | 131.5 (115.0, 156.8) | 135.0 (114.0, 156.0) | 0.019 |
| DBP, mmHg | 74.0 (63.0, 87.0) | 77.0 (67.0, 88.0) | 75.0 (63.0, 88.0) | 70.0 (62.2, 86.0) | 71.5 (61.8, 85.0) | 0.085 |
| Respiratory rate, bpm | 20.0 (16.0, 24.0) | 18.0 (15.0, 22.0) | 20.0 (17.0, 24.0) | 20.0 (16.0, 24.0) | 20.0 (18.0, 25.0) | 0.018 |
| Comorbidities, n (%) |  |  |  |  |  |  |
| Hypertensive | 145 (21.5) | 35 (20.7) | 30 (17.9) | 48 (28.6) | 32 (19) | 0.074 |
| Diabetes mellitus | 89 (13.2) | 21 (12.4) | 22 (13.1) | 27 (16.1) | 19 (11.3) | 0.611 |
| Chronic pulmonary disease | 18 ( 2.7) | 2 (1.2) | 6 (3.6) | 2 (1.2) | 8 (4.8) | 0.086 |
| Acute myocardial infarction | 28 ( 4.2) | 5 (3) | 6 (3.6) | 6 (3.6) | 11 (6.5) | 0.347 |
| Congestive heart failure | 40 ( 5.9) | 9 (5.3) | 11 (6.5) | 7 (4.2) | 13 (7.7) | 0.544 |
| Malignant tumor | 0 (0.0) | 0 (0.0) | 0 (0.0) | 0 (0.0) | 0 (0.0) | - |
| Chronic kidney disease | 40 ( 5.9) | 9 (5.3) | 11 (6.5) | 8 (4.8) | 12 (7.1) | 0.783 |
| Liver disease | 17 ( 2.5) | 2 (1.2) | 3 (1.8) | 4 (2.4) | 8 (4.8) | 0.225 |
| Laboratory tests |  |  |  |  |  |  |
| Platelets, K/uL | 209.0 (161.0, 268.8) | 214.5 (171.0, 268.8) | 209.0 (163.0, 252.0) | 211.0 (165.2, 265.2) | 200.0 (145.0, 276.5) | 0.646 |
| Haemoglobin, g/dL | 12.1 (10.0, 13.6) | 12.4 (10.5, 13.9) | 12.3 (9.9, 13.6) | 12.1 (10.0, 13.5) | 11.6 (9.4, 13.4) | 0.062 |
| White blood cells, K/uL | 10.2 (7.8, 13.9) | 9.0 (7.4, 11.6) | 10.1 (7.8, 13.3) | 11.2 (8.2, 14.6) | 10.8 (8.1, 16.0) | < 0.001 |
| Chloride, mmol/L | 106.0 (102.0, 109.0) | 106.0 (102.0, 108.0) | 106.0 (102.0, 109.0) | 105.0 (102.5, 108.0) | 105.0 (102.0, 109.0) | 0.854 |
| Calcium, mg/dL | 8.6 (8.2, 9.0) | 8.7 (8.4, 9.2) | 8.6 (8.2, 8.9) | 8.6 (8.1, 9.0) | 8.4 (8.0, 8.9) | 0.001 |
| Initial sodium, mEq/L | 139.0 (136.0, 141.0) | 139.0 (137.0, 141.0) | 139.0 (136.0, 141.0) | 139.0 (136.0, 141.0) | 139.0 (136.0, 142.0) | 0.772 |
| Initial potassium, mEq/L | 3.9 (3.6, 4.2) | 3.9 (3.7, 4.2) | 3.9 (3.6, 4.1) | 3.9 (3.5, 4.2) | 3.9 (3.5, 4.5) | 0.550 |
| Mean sodium, mEq/L | 139.8 (137.4, 142.3) | 139.2 (137.4, 140.7) | 139.8 (137.4, 142.0) | 140.1 (137.1, 143.0) | 140.2 (138.0, 143.9) | 0.003 |
| Mean potassium, mEq/L | 3.9 (3.7, 4.1) | 3.9 (3.7, 4.1) | 3.8 (3.7, 4.1) | 3.8 (3.7, 4.1) | 4.0 (3.7, 4.3) | < 0.001 |
| Creatinine, mg/dL | 106.0 (102.0, 109.0) | 106.0 (102.0, 108.0) | 106.0 (102.0, 109.0) | 105.0 (102.5, 108.0) | 105.0 (102.0, 109.0) | 0.839 |
| Glucose, mg/dL | 134.0 (110.0, 174.5) | 125.0 (106.0, 155.0) | 138.5 (112.5, 172.0) | 138.0 (110.5, 187.5) | 140.0 (115.0, 206.0) | < 0.001 |
| Intervention, n (%) |  |  |  |  |  |  |
| Haemodialysis | 7 ( 1.0) | 2 (1.2) | 1 (0.6) | 2 (1.2) | 2 (1.2) | 0.987 |
| IMV | 150 (22.3) | 14 (8.3) | 45 (26.8) | 44 (26.2) | 47 (28) | < 0.001 |
| Potassium supplementation | 140 (20.8) | 25 (14.8) | 38 (22.6) | 42 (25) | 35 (20.8) | 0.120 |
| Sodium supplementation | 294 (43.7) | 61 (36.1) | 85 (50.6) | 76 (45.2) | 72 (42.9) | 0.059 |
| Antiplatelet drugs | 227 (33.7) | 66 (39.1) | 52 (31) | 55 (32.7) | 54 (32.1) | 0.394 |
| Warfarin | 6 ( 0.9) | 1 (0.6) | 2 (1.2) | 2 (1.2) | 1 (0.6) | 0.872 |
| Furosemide | 48 ( 7.1) | 9 (5.3) | 11 (6.5) | 11 (6.5) | 17 (10.1) | 0.352 |
| Clinical Outcomes |  |  |  |  |  |  |
| 30-day mortality, n (%) | 109 (16.2) | 14 (8.3) | 27 (16.1) | 22 (13.1) | 46 (27.4) | < 0.001 |
| 90-day mortality, n (%) | 128 (19.0) | 18 (10.7) | 34 (20.2) | 26 (15.5) | 50 (29.8) | < 0.001 |
| 360-day mortality, n (%) | 151 (22.4) | 24 (14.2) | 38 (22.6) | 35 (20.8) | 54 (32.1) | < 0.001 |
| ICU length of stay, day | 4.0 (2.8, 6.8) | 3.1 (2.5, 4.2) | 4.3 (2.8, 6.7) | 5.0 (3.1, 8.4) | 5.0 (2.9, 9.5) | < 0.001 |
| Length of hospital stay, day | 8.8 (5.2, 15.9) | 6.1 (4.0, 10.3) | 8.5 (5.2, 14.8) | 9.8 (5.6, 16.9) | 11.0 (6.0, 21.0) | < 0.001 |

Abbreviations: SBP, systolic blood pressure; DBP, diastolic blood pressure; IMV, Invasive mechanical ventilation.

Supplementary Table S7. The associated between sodium and potassium variability and mortality in NWICU.

|  | **Sodium variability** | | | | **Potassium variability** | | | | |
| --- | --- | --- | --- | --- | --- | --- | --- | --- | --- |
|  | **Model I** | | **Model II** | | **Model I** | | **Model II** | | |
|  | **HR (95% CI)** | ***P*** | **HR (95% CI)** | ***P*** | **HR (95% CI)** | ***P*** | | **HR (95% CI)** | ***P*** |
| **30-day mortality** | | | | |  |  | |  |  |
| Continuous CV | 1.00 (0.99, 1.01) | 0.379 | 1.00 (0.99, 1.00) | 0.708 | 1.00 (1.00, 1.01) | 0.049 | | 1.00 (0.99, 1.01) | 0.134 |
| Quartiles of CV |  |  |  |  |  |  | |  |  |
| Q1 | *Reference* |  | *Reference* |  | *Reference* |  | | *Reference* |  |
| Q2 | 1.01 (0.47, 2.18) | 0.973 | 0.94 (0.43, 2.07) | 0.886 | 1.98 (1.04, 3.78) | 0.037 | | 1.15 (0.58, 2.26) | 0.685 |
| Q3 | 2.12 (1.09, 4.12) | 0.027 | 1.72 (0.86, 3.40) | 0.123 | 1.61 (0.82, 3.16) | 0.160 | | 1.04 (0.51, 2.11) | 0.907 |
| Q4 | 5.18 (2.84, 9.48) | < 0.001 | 4.04 (2.13, 7.63) | < 0.001 | 3.62 (1.99, 6.58) | < 0.001 | | 2.04 (1.06, 3.93) | 0.033 |
| **90-day mortality** | | | | |  |  | |  |  |
| Continuous CV | 1.00 (0.99, 1.00) | 0.472 | 1.00 (0.99, 1.00) | 0.785 | 1.00 (1.00, 1.00) | 0.081 | | 1.00 (0.99, 1.01) | 0.139 |
| Quartiles of CV |  |  |  |  |  |  | |  |  |
| Q1 | *Reference* |  | *Reference* |  | *Reference* |  | | *Reference* |  |
| Q2 | 1.33 (0.69, 2.55) | 0.389 | 1.21 (0.61, 2.36) | 0.586 | 1.97 (1.11, 3.49) | 0.020 | | 1.16 (0.63, 2.12) | 0.629 |
| Q3 | 2.00 (1.09, 3.67) | 0.025 | 1.65 (0.88, 3.10) | 0.113 | 1.49 (0.82, 2.72) | 0.189 | | 0.99 (0.53, 1.87) | 0.996 |
| Q4 | 4.63 (2.66, 8.03) | < 0.001 | 3.60 (2.00, 6.48) | < 0.001 | 3.13 (1.82, 5.31) | < 0.001 | | 1.77 (0.98, 3.23) | 0.059 |
| **360-day mortality** |  |  |  |  |  |  | |  |  |
| Continuous CV | 1.00 (0.99, 1.00) | 0.570 | 1.00 (0.99, 1.00) | 0.736 | 1.00 (0.99, 1.00) | 0.133 | | 1.00 (0.99, 1.01) | 0.116 |
| Quartiles of CV |  |  |  |  |  |  | |  |  |
| Q1 | *Reference* |  | *Reference* |  | *Reference* |  | | *Reference* |  |
| Q2 | 1.10 (0.63, 1.95) | 0.731 | 0.97 (0.53, 1.74) | 0.912 | 1.66 (1.00, 2.78) | 0.050 | | 1.09 (0.64, 1.87) | 0.729 |
| Q3 | 1.63 (0.97, 2.76) | 0.067 | 1.41 (0.82, 2.41) | 0.219 | 1.51 (0.92, 2.55) | 0.116 | | 1.16 (0.67, 2.00) | 0.591 |
| Q4 | 3.68 (2.29, 5.91) | < 0.001 | 3.21 (1.93, 5.33) | <0.001 | 2.57 (1.59, 4.17) | < 0.001 | | 1.67 (0.98, 2.85) | 0.058 |

Sodium variability: Q1 < 0.95; 0.95 ≤ Q2 <1.50; 1.50 ≤ Q3 < 2.36; Q4 ≥ 2.36;

Potassium variability: Q1 < 5.18; 5.18 ≤ Q2 < 7.90; 7.90 ≤ Q3 < 10.88; Q4 ≥ 10.88.

Model I: Unadjusted;

Model II: Adjusted by Age, Male, Race, Heart rate, Systolic blood pressure, Diastolic blood pressure, Respiratory rate, Hypertensive, Diabetes mellitus, Chronic pulmonary disease, Acute myocardial infarction, Congestive heart failure, Chronic kidney disease, Liver disease, Platelets, Haemoglobin, White blood cells, Chloride, Calcium, Creatinine, Glucose, Haemodialysis, Invasive mechanical ventilation, Potassium supplementation, Sodium supplementation, Antiplatelet drugs, Warfarin, Furosemide.

Abbreviations: HR, hazard ratio; CI, confidence interval.

Supplementary Table S8. The associated between combined sodium-potassium variability and mortality in NWICU.

|  | **Model I** | | **Model II** | | |
| --- | --- | --- | --- | --- | --- |
|  | **HR (95% CI)** | ***P*** | **HR (95% CI)** | ***P*** | |
| **30-day mortality** | | | | |  |
| Na^+^ CV < 1.97 & K^+^ CV < 11.78 | *reference* |  | *reference* |  | |
| Na^+^ CV < 1.97 & K^+^ CV > 11.78 | 2.53 (1.19, 5.39) | 0.016 | 2.07 (0.93, 4.58) | 0.071 | |
| Na^+^ CV > 1.97 & K^+^ CV < 11.78 | 4.70 (2.91, 7.62) | < 0.001 | 3.28 (1.97, 5.45) | < 0.001 | |
| Na^+^ CV > 1.97 & K^+^ CV > 11.78 | 6.72 (3.99, 11.32) | < 0.001 | 5.42 (3.08, 9.53) | < 0.001 | |
| **90-day mortality** | | | | |  |
| Na^+^ CV < 1.97 & K^+^ CV < 11.66 | *reference* |  | *reference* |  | |
| Na^+^ CV < 1.97 & K^+^ CV > 11.66 | 1.96 (0.97, 3.94) | 0.057 | 1.67 (0.80, 3.48) | 0.171 | |
| Na^+^ CV > 1.97 & K^+^ CV < 11.66 | 3.78 (2.46, 5.80) | < 0.001 | 2.75 (1.75, 4.33) | < 0.001 | |
| Na^+^ CV > 1.97 & K^+^ CV > 11.66 | 5.26 (3.29, 8.39) | < 0.001 | 4.32 (2.57, 7.26) | < 0.001 | |
| **360-day mortality** |  |  |  |  | |
| Na^+^ CV < 1.97 & K^+^ CV < 9.67 | *reference* |  | *reference* |  | |
| Na^+^ CV < 1.97 & K^+^ CV > 9.67 | 2.12 (1.26, 3.56) | 0.004 | 1.75 (1.00, 3.05) | 0.048 | |
| Na^+^ CV > 1.97 & K^+^ CV < 9.67 | 4.34 (2.80, 6.72) | < 0.001 | 3.34 (2.09, 5.33) | < 0.001 | |
| Na^+^ CV > 1.97 & K^+^ CV > 9.67 | 4.36 (2.84, 6.69) | < 0.001 | 4.05 (2.54, 6.47) | < 0.001 | |

Model I: Unadjusted;

Model II: Adjusted by Age, Male, Race, Heart rate, Systolic blood pressure, Diastolic blood pressure, Respiratory rate, Hypertensive, Diabetes mellitus, Chronic pulmonary disease, Acute myocardial infarction, Congestive heart failure, Chronic kidney disease, Liver disease, Platelets, Haemoglobin, White blood cells, Chloride, Calcium, Creatinine, Glucose, Haemodialysis, Invasive mechanical ventilation, Potassium supplementation, Sodium supplementation, Antiplatelet drugs, Warfarin, Furosemide.

Abbreviations: HR, hazard ratio; CI, confidence interval.

Supplementary Table S9. Sensitivity analysis of associated between mean sodium and potassium and 30/90/360-day mortality in MIMIC-IV and NWICU.

|  | **MIMIC-IV** | | | | **NWICU** | | | | | | | | | |
| --- | --- | --- | --- | --- | --- | --- | --- | --- | --- | --- | --- | --- | --- | --- |
|  | **Mean Sodium** | | **Mean Potassium** | | **Mean Sodium** | | | | **Mean Potassium** | | | | | |
|  | **HR (95% CI)** | ***P*** | **HR (95% CI)** | ***P*** | **HR (95% CI)** | | ***P*** | | | **HR (95% CI)** | | ***P*** | | |
| **30-day mortality** | | | | | |  | |  | | |  | |  |  |
| Norm-group | *reference* |  | *reference* |  | *reference* | |  | | | *reference* | |  | | |
| Hypo-group | 1.38 (1.02 1.98) | 0.042 | 1.01 (0.68, 1.49) | 0.951 | 1.11 (0.47, 2.57) | | 0.806 | | | 1.32 (0.70, 2.48) | | 0.379 | | |
| Hyper-group | 1.56 (1.12, 1.85) | 0.003 | 2.27 (1.16, 4.46) | 0.017 | 4.48 (2.89, 6.95) | | < 0.001 | | | 2.38 (0.72, 7.85) | | 0.152 | | |
| **90-day mortality** | | | | | |  | |  | | |  | |  |  |
| Norm-group | *reference* |  | *reference* |  | *reference* | |  | | | *reference* | |  | | |
| Hypo-group | 1.34 (1.01, 1.79) | 0.045 | 0.96 (0.68, 1.35) | 0.814 | 1.23 (0.60, 2.51) | | 0.567 | | | 1.11 (0.59, 2.06) | | 0.745 | | |
| Hyper-group | 1.43 (1.06, 1.94) | 0.018 | 2.18 (1.16, 4.12) | 0.016 | 3.81 (2.51, 5.76) | | < 0.001 | | | 2.96 (1.05, 8.39) | | 0.040 | | |
| **360-day mortality** |  |  |  |  |  | |  | | |  | |  | | |
| Norm-group | *reference* |  | *reference* |  | *reference* | |  | | | *reference* | |  | | |
| Hypo-group | 1.32 (1.01, 1.81) | 0.044 | 1.05 (0.78, 1.41) | 0.748 | 0.99 (0.49, 1.99) | | 0.982 | | | 1.11 (0.63, 1.97) | | 0.710 | | |
| Hyper-group | 1.38 (1.05, 1.81) | 0.023 | 2.36 (1.29, 4.32) | 0.005 | 3.50 (2.37, 5.15) | | < 0.001 | | | 3.78 (1.08, 7.86) | | 0.042 | | |

Mean Sodium: Hypo-group: NA^+^ <135mmol/L, Norm-group: 135mmol/L≤ NA^+^≤ 145mmol/L, Hyper-group: NA^+^>145mmol/L;

Mean Potassium: Hypo-group: K^+^ <3.5mmol/L, Norm-group: 3.5mmol/L≤K^+^≤ 5.5mmol/L, Hyper-group: K^+^>5.5mmol/L;

MIMIC-IV: Model: Adjusted by Age, Male, Race, Heart rate, Systolic blood pressure, Diastolic blood pressure, Respiratory rate, Hypertensive, Diabetes mellitus, Chronic pulmonary disease, Acute myocardial infarction, Congestive heart failure, Malignant tumor, Chronic kidney disease, Liver disease, Platelets, Haemoglobin, White blood cells, Chloride, Calcium, Creatinine, Glucose, Acute Physiology Score III, Glasgow Coma Scale, Renal Replacement Therapy, Mechanical ventilation, Potassium supplementation, Sodium supplementation, Thrombolysis, Antiplatelet drugs, Warfarin, Furosemide.

NWICU: Model: Adjusted by Age, Male, Race, Heart rate, Systolic blood pressure, Diastolic blood pressure, Respiratory rate, Hypertensive, Diabetes mellitus, Chronic pulmonary disease, Acute myocardial infarction, Congestive heart failure, Chronic kidney disease, Liver disease, Platelets, Haemoglobin, White blood cells, Chloride, Calcium, Creatinine, Glucose, Haemodialysis, Invasive mechanical ventilation, Potassium supplementation, Sodium supplementation, Antiplatelet drugs, Warfarin, Furosemide.

Abbreviations: HR, hazard ratio; CI, confidence interval.

Supplementary Table S10. Sensitivity analysis of association between sodium and potassium variability and ICU/Hospital mortality in MIMIC-IV and NWICU.

|  | **MIMIC-IV** | | | | **NWICU** | | | |
| --- | --- | --- | --- | --- | --- | --- | --- | --- |
|  | **Hospital mortality** | | **ICU mortality** | | **Hospital mortality** | | **ICU mortality** | |
|  | **HR (95% CI)** | ***P*** | **HR (95% CI)** | ***P*** | **HR (95% CI)** | ***P*** | **HR (95% CI)** | ***P*** |
| **Sodium variability** |  |  |  |  |  |  |  |  |
| Continuous Sodium variability | 1.22 (1.15, 1.30) | < 0.001 | 1.28 (1.18, 1.37) | < 0.001 | 1.00 (0.99, 1.01) | 0.567 | 1.00 (0.99, 1.00) | 0.974 |
| Quartiles of Sodium variability |  |  |  |  |  |  |  |  |
| Q1 | *Reference* |  | *Reference* |  | *Reference* |  | *Reference* |  |
| Q2 | 1.06 (0.77, 1.46) | 0.694 | 1.04 (0.70, 1.55) | 0.838 | 1.58 (0.53, 4.71) | 0.410 | 1.53 (0.40, 5.81) | 0.530 |
| Q3 | 1.44 (1.07, 1.95) | 0.017 | 1.44 (0.99, 2.09) | 0.056 | 2.29 (0.84, 6.23) | 0.105 | 1.65 (0.45, 5.97) | 0.449 |
| Q4 | 1.52 (1.13, 2.04) | 0.006 | 1.69 (1.18, 2.42) | 0.004 | 4.00 (1.55, 10.36) | 0.004 | 2.05 (0.59, 7.09) | 0.255 |
| **Potassium variability** |  |  |  |  |  |  |  |  |
| Continuous Potassium variability | 1.02 (1.01, 1.04) | 0.006 | 1.03 (1.01, 1.05) | < 0.001 | 1.00 (0.99, 1.01) | 0.570 | 1.00 (0.99, 1.00) | 0.711 |
| Quartiles of Potassium variability |  |  |  |  |  |  |  |  |
| Q1 | *Reference* |  | *Reference* |  | *Reference* |  | *Reference* |  |
| Q2 | 1.06 (0.79, 1.42) | 0.700 | 1.11 (0.76, 1.60) | 0.585 | 0.64 (0.28, 1.45) | 0.292 | 0.76 (0.25, 2.34) | 0.639 |
| Q3 | 1.04 (0.77, 1.38) | 0.805 | 1.12 (0.70, 1.47) | 0.924 | 0.71 (0.32, 1.59) | 0.411 | 0.67 (0.22, 2.00) | 0.477 |
| Q4 | 1.37 (1.03, 1.81) | 0.029 | 1.69 (1.19, 2.40) | 0.003 | 1.01 (0.47, 2.15) | 0.980 | 1.23 (0.43, 3.53) | 0.691 |

MIMIC-IV: Sodium variability: Q1< 0.94; 0.94 ≤ Q2 < 1.43; 1.43 ≤ Q3 < 2.04; Q4 ≥ 2.04; Potassium variability: Q1 < 5.25; 5.25 ≤ Q2 < 7.90; 7.90 ≤ Q3 < 11.62; Q4 ≥ 11.62.

Model: Adjusted by Age, Male, Race, Heart rate, Systolic blood pressure, Diastolic blood pressure, Respiratory rate, Hypertensive, Diabetes mellitus, Chronic pulmonary disease, Acute myocardial infarction, Congestive heart failure, Malignant tumor, Chronic kidney disease, Liver disease, Platelets, Haemoglobin, White blood cells, Chloride, Calcium, Creatinine, Glucose, Acute Physiology Score III, Glasgow Coma Scale, Renal Replacement Therapy, Mechanical ventilation, Potassium supplementation, Sodium supplementation, Thrombolysis, Antiplatelet drugs, Warfarin, Furosemide.

NWICU: Sodium variability: Q1 < 0.95; 0.95 ≤ Q2 < 1.50; 1.50 ≤ Q3 < 2.36; Q4 ≥ 2.36; Potassium variability: Q1 <5.18; 5.18 ≤ Q2 < 7.90; 7.90 ≤ Q3 < 10.88; Q4 ≥ 10.88.

Model: Adjusted by Age, Male, Race, Heart rate, Systolic blood pressure, Diastolic blood pressure, Respiratory rate, Hypertensive, Diabetes mellitus, Chronic pulmonary disease, Acute myocardial infarction, Congestive heart failure, Chronic kidney disease, Liver disease, Platelets, Haemoglobin, White blood cells, Chloride, Calcium, Creatinine, Glucose, Haemodialysis, Invasive mechanical ventilation, Potassium supplementation, Sodium supplementation, Antiplatelet drugs, Warfarin, Furosemide.

Abbreviations: HR, hazard ratio; CI, confidence interval; ICU, intensive care unit.

Supplementary Table S11. Sensitivity analysis of association between sodium and potassium variability and mortality after excluding patients with tumours in MIMIC-IV.

|  | **Sodium variability** | | **Potassium variability** | |
| --- | --- | --- | --- | --- |
|  | **HR (95% CI)** | ***P*** | **HR (95% CI)** | ***P*** |
| **30-day mortality** |  |  |  |  |
| Continuous CV | 1.24 (1.18, 1.31) | < 0.001 | 1.03 (1.02, 1.05) | < 0.001 |
| Quartiles of CV |  |  |  |  |
| Q1 | *reference* |  | *reference* |  |
| Q2 | 1.10 (0.82, 1.49) | 0.528 | 1.15 (0.87, 1.51) | 0.336 |
| Q3 | 1.48 (1.11, 1.97) | 0.008 | 1.00 (0.76, 1.33) | 0.982 |
| Q4 | 1.99 (1.51, 2.61) | < 0.001 | 1.52 (1.16, 1.98) | 0.002 |
| **90-day mortality** |  |  |  |  |
| Continuous CV | 1.21 (1.15, 1.27) | < 0.001 | 1.03 (1.01, 1.04) | < 0.001 |
| Quartiles of CV |  |  |  |  |
| Q1 | *reference* |  | *reference* |  |
| Q2 | 0.98 (0.76, 1.27) | 0.887 | 1.03 (0.81, 1.31) | 0.810 |
| Q3 | 1.32 (1.04, 1.69) | 0.025 | 0.95 (0.74, 1.21) | 0.675 |
| Q4 | 1.67 (1.32, 2.12) | < 0.001 | 1.36 (1.08, 1.72) | 0.009 |
| **360-day mortality** |  |  |  |  |
| Continuous CV | 1.63 (1.31, 2.02) | < 0.001 | 1.02 (1.01, 1.04) | < 0.001 |
| Quartiles of CV |  |  |  |  |
| Q1 | *reference* |  | *reference* |  |
| Q2 | 1.02 (0.81, 1.29) | 0.842 | 0.56 (0.14, 2.20) | 0.395 |
| Q3 | 1.30 (1.04, 1.62) | 0.022 | 1.02 (1.01, 1.04) | 0.001 |
| Q4 | 1.63 (1.31, 2.02) | < 0.001 | 1.52 (1.18, 1.96) | < 0.001 |

Sodium variability: Q1< 0.94; 0.94 ≤ Q2 < 1.43; 1.43 ≤ Q3 < 2.04; Q4 ≥ 2.04.

Potassium variability: Q1 < 5.25; 5.25 ≤ Q2 < 7.90; 7.90 ≤ Q3 < 11.62; Q4 ≥ 11.62.

Model: Adjusted by Age, Male, Race, Heart rate, Systolic blood pressure, Diastolic blood pressure, Respiratory rate, Hypertensive, Diabetes mellitus, Chronic pulmonary disease, Acute myocardial infarction, Congestive heart failure, Chronic kidney disease, Liver disease, Platelets, Haemoglobin, White blood cells, Chloride, Calcium, Creatinine, Glucose, Haemodialysis, Invasive mechanical ventilation, Potassium supplementation, Sodium supplementation, Antiplatelet drugs, Warfarin, Furosemide.

Abbreviations: HR, hazard ratio; CI, confidence interval; CV, coefficient of variation.

Supplementary Table S12. Sensitivity analysis of the association between sodium and potassium variability and mortality after extensive adjustment for intensive care interventions and metabolic confounders.

|  | **MIMIC-IV** | | | | **NWICU** | | | | |
| --- | --- | --- | --- | --- | --- | --- | --- | --- | --- |
|  | **Sodium variability** | | **Potassium variability** | | **Sodium variability** | | **Potassium variability** | | |
|  | **HR (95% CI)** | ***P*** | **HR (95% CI)** | ***P*** | **HR (95% CI)** | ***P*** | | **HR (95% CI)** | ***P*** |
| **30-day mortality** | | | | |  |  | |  |  |
| Continuous CV | 1.16 (1.09, 1.23) | < 0.001 | 1.03 (1.01, 1.04) | < 0.001 | 1.09 (1.02, 1.15) | < 0.001 | | 1.02 (1.01, 1.04) | 0.010 |
| Quartiles of CV |  |  |  |  |  |  | |  |  |
| Q1 | *Reference* |  | *Reference* |  | *Reference* |  | | *Reference* |  |
| Q2 | 1.06 (0.81, 1.39) | 0.666 | 1.03 (0.79, 1.32) | 0.829 | 0.98 (0.77, 1.00) | 0.125 | | 0.92 (0.75, 1.16) | 0.360 |
| Q3 | 1.44 (1.12, 1.86) | 0.005 | 1.05 (0.81, 1.35) | 0.719 | 1.20 (0.98, 1.51) | 0.365 | | 1.02 (0.80, 1.31) | 0.607 |
| Q4 | 1.53 (1.17, 1.99) | 0.002 | 1.34 (1.05, 1.71) | 0.018 | 1.35 (1.09, 1.69) | 0.006 | | 1.14 (1.05, 1.23) | 0.023 |
| **90-day mortality** | | | | |  |  | |  |  |
| Continuous CV | 1.13 (1.07, 1.20) | < 0.001 | 1.02 (1.01, 1.03) | < 0.001 | 1.09 (1.02, 1.20) | 0.036 | | 1.01 (0.99, 1.02) | 0.103 |
| Quartiles of CV |  |  |  |  |  |  | |  |  |
| Q1 | *Reference* |  | *Reference* |  | *Reference* |  | | *Reference* |  |
| Q2 | 0.95 (0.75, 1.19) | 0.641 | 0.90 (0.72, 1.12) | 0.351 | 0.97 (0.76, 1.20) | 0.621 | | 0.91 (0.73, 1.12) | 0.412 |
| Q3 | 1.27 (1.03, 1.58) | 0.029 | 0.96 (0.77, 1.19) | 0.713 | 1.03 (0.89, 1.22) | 0.289 | | 0.99 (0.79, 1.17) | 0.326 |
| Q4 | 1.33 (1.06, 1.66) | 0.014 | 1.26 (1.02, 1.56) | 0.029 | 1.23 (1.02, 1.34) | 0.025 | | 1.17 (1.02, 1.33) | 0.038 |
| **360-day mortality** |  |  |  |  |  |  | |  |  |
| Continuous CV | 1.11 (1.06, 1.18) | < 0.001 | 1.02 (1.01, 1.03) | < 0.001 | 1.04 (0.98, 1.12) | 0.068 | | 1.00 (0.98, 1.01) | 0.129 |
| Quartiles of CV |  |  |  |  |  |  | |  |  |
| Q1 | *Reference* |  | *Reference* |  | *Reference* |  | | *Reference* |  |
| Q2 | 0.98 (0.80, 1.21) | 0.896 | 0.95 (0.78, 1.16) | 0.629 | 0.88 (0.53, 1.13) | 0.213 | | 0.82 (0.54, 1.07) | 0.635 |
| Q3 | 1.28 (1.05, 1.56) | 0.013 | 0.94 (0.77, 1.15) | 0.546 | 1.01 (0.85, 1.20) | 0.416 | | 0.91 (0.68, 1.16) | 0.231 |
| Q4 | 1.33 (1.08, 1.63) | 0.006 | 1.25 (1.03, 1.52) | 0.022 | 1.21 (1.09, 1.30) | 0.045 | | 1.15 (1.01, 1.25) | 0.028 |

Quartile cut-offs for sodium and potassium variability in both cohorts were defined identically to those in the primary analysis.

Model was adjusted by Age, Male, Race, Heart rate, Systolic blood pressure, Diastolic blood pressure, Respiratory rate, Hypertensive, Diabetes mellitus, Chronic pulmonary disease, Acute myocardial infarction, Congestive heart failure, Malignant tumor, Chronic kidney disease, Liver disease, Platelets, Haemoglobin, White blood cells, Chloride, Calcium, Creatinine, Glucose, Acute Physiology Score III, Glasgow Coma Scale, Renal Replacement Therapy, Mechanical ventilation, Potassium supplementation, Sodium supplementation, Thrombolysis, Antiplatelet drugs, Warfarin, Furosemide, 24-hour net fluid balance, calculated serum osmolarity, glucose coefficient of variation, insulin, sepsis, vasopressor, pH, and lactate.

Abbreviations: HR, hazard ratio; CI, confidence interval; CV, coefficient of variation.

Supplementary Figure S1. Flowchart of the study population. MIMIC-IV, Medical Information Mart for Intensive Care; NWICU, Northwestern ICU Database; ICU, intensive care unit, CV, Coefficient of variation.

Supplementary Figure S2. Kaplan-Meier survival curves between sodium and potassium variability and 30-day, 90-day, 360-day mortality in NWICU. HR, hazard ratio; CI, confidence interval.

Supplementary Figure S3. The restricted cubic spline for the relationships between sodium and potassium variability and 30-day, 90-day, 360-day mortality in NWICU. HR, hazard ratio; CI, confidence interval.

Supplementary Figure S4. Kaplan-Meier survival curves between combined sodium-potassium variability and 30-day, 90-day, 360-day mortality in MIMIC-IV (a, b, c) and NWICU (d, e, f). MIMIC-IV: 30-day mortality: G1: Na+ CV < 1.54 & K+ CV < 15.17; G2: Na+ CV < 1.54 & K+ CV > 15.17; G3: Na+ CV > 1.54 & K+ CV < 15.17; G4: Na+ CV > 1.54 & K+ CV > 15.17; 90-day mortality: Na+ CV < 1.54 & K+ CV < 11.59; G2: Na+ CV < 1.54 & K+ CV > 11.59; G3: Na+ CV > 1.54 & K+ CV < 11.59; G4: Na+ CV > 1.54 & K+ CV > 11.59; 360-day mortality: Na+ CV < 1.58 & K+ CV < 11.59; G2: Na+ CV < 1.58 & K+ CV > 11.59; G3: Na+ CV > 1.58 & K+ CV < 11.59; G4: Na+ CV > 1.58 & K+ CV > 11.59. NWICU: 30-day mortality: G1: Na+ CV < 1.97 & K+ CV < 11.78; G2: Na+ CV < 1.97 & K+ CV > 11.78; G3: Na+ CV > 1.97 & K+ CV < 11.78; G4: Na+ CV > 1.97 & K+ CV > 11.78; 90-day mortality: Na+ CV < 1.97 & K+ CV < 11.66; G2: Na+ CV < 1.97 & K+ CV > 11.66; G3: Na+ CV > 1.97 & K+ CV < 11.66; G4: Na+ CV > 1.97 & K+ CV > 11.66; 360-day mortality: Na+ CV < 1.97 & K+ CV < 9.67; G2: Na+ CV < 1.97 & K+ CV > 9.67; G3: Na+ CV > 1.97 & K+ CV < 9.67; G4: Na+ CV > 1.97 & K+ CV > 9.67.

Supplementary Figure S5. Determination of optimal risk stratification cut-off points for sodium and potassium variability on 30-day, 90-day, 360-day mortality in NWICU.
